# Neonatal-Inspired Reprogramming of Microglial Pan-Programmed Cell Death Enhances Regeneration in Adult Spinal Cord Injury

**DOI:** 10.34133/research.0759

**Published:** 2025-07-02

**Authors:** Beibei Yu, Yongfeng Zhang, Yujie Yang, Shijie Yang, Haining Wu, Xue Gao, Yiming Hao, Shengyou Li, Bing Xia, Jintao Liu, Lingli Guo, Borui Xue, Mingze Qin, Huangtao Chen, Jianzhong Li, Shouping Gong, Teng Ma, Jinghui Huang

**Affiliations:** ^1^ Department of Orthopaedics, Xijing Hospital, Fourth Military Medical University, Xi’an 710032, China.; ^2^ Department of Neurosurgery, The Second Affiliated Hospital, Xi’an Jiaotong University, Xi’an 710004, China.; ^3^ Department of Orthopedics, Naval Hospital of Eastern Theater, Zhoushan 316000, China.; ^4^ Department of Thoracic Surgery, Second Affiliated Hospital of Xi’an Jiao Tong University, Xi’an, China.; ^5^ Xi’an Medical University, Xi’an, China.; ^6^ Military Medical Innovation Center, Fourth Military Medical University, Xi’an 710032, China.

## Abstract

In adult mammals, programmed cell death (PCD) facilitates tissue remodeling and regeneration in spinal cord injury (SCI), but excessive activation impedes SCI repair. However, no comprehensive pan-PCD atlas exists that encompasses diverse cell death patterns to fully elucidate PCD in adult SCI and develop strategies for modulating the excessive PCD response. Here, we identified neonatal mice with balanced PCD post-SCI as an ideal model for adult SCI. Accordingly, we developed “Thanatoset”, an SCI-specific gene panel to map tissue and cellular pan-PCD dynamics across neonatal and adult mice. Microglia were identified as pivotal mediators of pan-PCD, showing greater vulnerability in adults than in neonates. According to computational drug screening, withaferin A can revert microglial pan-PCD in adults to a neonatal-like regenerative state. Histological, functional, and molecular analyses corroborated that withaferin A enhances SCI recovery in adults by modulating microglial pan-PCD. These findings highlight the therapeutic potential of the pan-PCD framework for developing strategies to restore regeneration and improve SCI outcomes.

## Introduction

Spinal cord injury (SCI), a debilitating neurological condition affecting approximately 0.9 million individuals annually, contributes to 6.2 million disability-adjusted life years worldwide, which reflects its profound socioeconomic and health impacts [1,2]. Despite marked advances in medical interventions and rehabilitation strategies, SCI remains a leading cause of long-term functional impairment, with many patients experiencing irreversible disabilities [3,4]. The complex pathology of SCI, characterized by multifaceted processes including inflammation, cell death, and glial scar formation, presents substantial challenges for functional recovery and limits the efficacy of current treatments [5]. Thus, innovative therapeutic approaches are required.

Programmed cell death (PCD), which include apoptosis, necroptosis, pyroptosis, ferroptosis, and autophagy, plays a crucial role in determining long-term recovery following SCI [6,7]. Although physiologically regulated PCD serves essential functions, its excessive activation impairs spinal cord repair by causing excessive neuronal loss, exacerbating inflammation, and inducing secondary damage through oxidative stress, ultimately transforming protective mechanisms into destructive processes [5,8]. PCD mechanisms are numerous and closely connected, with substantial cross-talk and regulatory interactions among different cell death modalities [9,10]. However, current SCI research has largely taken a compartmentalized approach, focusing on individual PCD subtypes while neglecting the complex network of interactions that collectively shape the cellular response to injury. Given that PCD exhibits notable tissue specificity and disease relevance, this reductionist approach limits our comprehensive understanding of SCI pathology [8,11–13]. Accordingly, establishing an SCI-specific framework that integrates multiple cell death patterns (pan-PCD) could elucidate how dysregulated PCD drives functional decline and provide a foundation for developing targeted therapeutic interventions to modulate PCD, thereby improving outcomes for patients with SCI.

PCD has long been recognized as an evolutionarily conserved regulatory mechanism essential for tissue homeostasis and repair [14–17]. Adult mammals exhibit dysregulated PCD following tissue injury; however, organisms with complete regenerative abilities, such as axolotls and zebrafish, demonstrate a finely tuned level of PCD [18,19]. This controlled PCD facilitates the efficient removal of damaged and necrotic cells, regulates inflammatory cascades, and orchestrates tissue remodeling, suggesting that balanced PCD levels may enhance tissue regeneration [18–20]. However, the PCD profile of these regenerative species cannot be directly extrapolated to adult mammals because of evolutionary divergences, including accumulated genetic mutations, regulatory network changes, and genome reorganization [21,22]. Consequently, studying intraspecies mammalian models with enhanced regenerative potential may provide more clinically relevant insights [23]. Recent advances have revealed marked age-dependent variations in mammalian regeneration, with neonatal mice exhibiting exceptional regenerative capacity across multiple organs, including the heart, skin, brain, lungs, and spinal cord [24–28]. Notably, neonatal mammals can fully regenerate their spinal cords, restoring both structural and functional integrity; therefore, their pan-PCD regulation may represent an optimal reference for adult SCI models [28,29]. However, 2 critical challenges remain: the precise quantification of pan-PCD dynamics following SCI and the development of strategies to modulate adult pan-PCD states to replicate the optimal regenerative conditions observed in neonates.

In this study, we demonstrated that neonatal mice maintain “optimal” PCD levels that are conducive to neuroregeneration, in contrast to the pathological overactivation of PCD observed in adult mice following SCI. To comprehensively characterize this differential response, we developed Thanatoset, a novel gene tool specifically designed to capture pan-PCD dynamics during the acute injury response window [1 to 7 d post-injury (dpi)] at both tissue and cellular levels following SCI. Our findings revealed that microglia, the principal immune regulators in SCI pathology, display increased susceptibility to pan-PCD in adult SCI, with microglial responses exhibiting marked age-dependent variations. Through comprehensive virtual drug screening of pharmacological compounds, we identified withaferin A (WFA) as a potent agent capable of reprogramming adult microglial pan-PCD toward a regenerative neonatal-like state. Mechanistically, WFA effectively ameliorated the inflammatory microenvironment and enhanced neuroregenerative capacity in both in vitro microglial cultures and in vivo adult SCI models by regulating the nuclear factor κB (NF-κB) signaling pathway. These findings establish a novel therapeutic approach that utilizes the age-dependent modulation of pan-PCD to restore neural homeostasis and improve functional outcomes in patients with SCI.

## Results

### Neonatal mice with balanced PCD activation serve as a model for adult SCI recovery

**Fig. 1.**
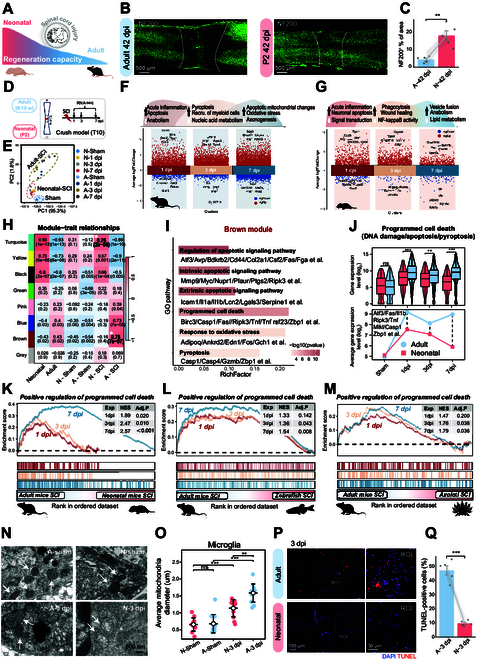
Bulk transcriptomic analysis reveals enhanced regenerative capacity correlates with optimal PCD response. (A) Schematic representation of spinal cord regeneration capacity following injury in neonatal and adult mice. (B) Representative NF200 staining images of spinal cord sections at 42 d post-injury (dpi) showing enhanced axonal regrowth in neonatal compared to adult mice. (C) Quantification of neurofilament (NF) immunofluorescence intensity in lesion core regions (*n* = 5). (D) Experimental workflow for bulk RNA sequencing. R and C indicate rostral and caudal regions, respectively. (E) Principal components analysis (PCA) plot showing distinct clustering of samples by injury phase and age group. (F and G) Volcano plots showing differentially expressed genes (DEGs) in adult (F) and neonatal (G) spinal cords at 1, 3, and 7 dpi compared to sham condition. DEGs were defined by log_2_|FC| > 1.5 and *P* < 0.05. Gene ontology (GO) terms with *P* < 0.01 are highlighted in the upper panel. (H) Module–trait correlation heatmap from WGCNA, with bar values representing the correlation between different groups and gene modules. (I) GO enrichment pathway of the brown module (*P* < 0.01). (J) Average expression of PCD-associated genes in the turquoise module across time points (top) and comparison between neonatal and adult mice (bottom). (K to M) Gene Set Enrichment Analysis (GSEA) for “positive regulation of PCD” terms comparing post-SCI responses at 1, 3, and 7 dpi between (K) adult versus neonatal mice, (L) adult mice versus adult zebrafish, and (M) adult mice versus adult axolotls. (N) Representative TEM images at 3 dpi showing mitochondrial morphology in microglia from lesion areas of neonatal and adult mice. (O) Quantification of mitochondrial diameter in microglia in lesion area (*n* = 12). (P) Representative TUNEL staining images of lesion rims at 3 dpi in adult and neonatal mice. (Q) Quantification of TUNEL-positive cells per section in lesion rim (*n* = 5). Statistical analysis was conducted using a 2-tailed *t* test (C and Q), Wilcoxon rank-sum test (J), and one-way ANOVA with Tukey (O). ns = not significant, **P < 0.01, ***P < 0.001.

To systematically examine age-dependent regenerative capacity following SCI, we established T10 crush SCI models in both neonatal mice [postnatal day 2 (P2)] and adult mice (8 to 10 weeks old) (Fig. 1A and Fig. S1A). At 42 dpi, a remarkably larger area of neurofilament (NF200)-positive regenerated axons was observed in the injured spinal cords of neonatal mice than in those of adult mice (Fig. 1B and C). Correspondingly, neonatal mice exhibited superior hindlimb motor function recovery relative to adults, as demonstrated by the CatWalk analysis (Fig. S1B to E).

To delineate the molecular mechanisms driving these phenotypic differences, we performed bulk transcriptomic sequencing at multiple time points (sham, 1, 3, and 7 dpi) (Fig. 1D and E and Fig. S1F and G). A greater number of differentially expressed genes (DEGs) were identified in adult mice relative to neonatal mice following SCI when compared to sham conditions (Fig. S1H). Gene Ontology (GO) analysis showed that up-regulated DEGs in adults were associated with acute inflammatory responses, immune cell recruitment, and oxidative stress pathways at 1 to 7 dpi, whereas axonal regeneration pathways were down-regulated (Fig. 1F). In contrast, neonatal mice exhibited marked up-regulation of tissue repair-related pathways, including wound healing, anabolic processes, and suppression of NF-κB inflammatory signaling [30], particularly at 3 dpi (Fig. 1G). Notably, we observed distinct temporal patterns in PCD regulation: Apoptosis and pyroptosis remained persistently active in adult mice throughout 1 to 7 dpi, whereas their enrichment in neonatal mice showed substantial attenuation by 3 to 7 dpi (Fig. 1F and G).

We next employed weighted gene coexpression network analysis (WGCNA) to identify the key biological pathways distinguishing adult and neonatal SCI responses and identified 8 distinct gene modules (Fig. 1H). Among these, the turquoise module, which showed a strong correlation (Cor) with neonatal SCI (Cor = 0.88, *P* < 0.001), was associated with neural regeneration pathways (Fig. S2A and B). Core regulatory genes within this module, such as *Gh*, *Grm5*, *Lingo1*, and *Hes5*, maintained sustained elevated expression throughout the injury phase in neonates but showed a progressive decline in adults [31,32] (Fig. S2C). Conversely, the brown module, which had the strongest correlation with adult SCI (Cor = 0.93, *P* < 0.001), was enriched in multiple PCD pathways, particularly apoptosis, pyroptosis, and DNA damage (Fig. 1I and Fig. S2D). Notably, key PCD-related genes, such as *Atf3*, *Fas*, *Il1b*, *Ripk3*, and *Tnf*, displayed comparable baseline expression levels in both age groups under physiological conditions [33,34] (Fig. 1J). However, the expression of these genes was markedly elevated in adult mice at 1 to 7 dpi, reflecting an amplified injury response and persistent activation of cell death pathways in adults.

We next conducted a pairwise differential analysis to directly compare the injury responses between adult and neonatal mice, identifying 2,749 DEGs at 1 dpi, 2,042 DEGs at 3 dpi, and 2,328 DEGs at 7 dpi in adult mice (Fig. S2E). Gene Set Enrichment Analysis (GSEA) across 1 to 7 dpi revealed persistent up-regulation of PCD-related pathways, immune and inflammatory responses, and gliogenesis in adult mice, in contrast to those in neonatal mice (Fig. S2F to H). Neuroprotection and regeneration pathways were concurrently down-regulated in adults (Fig. S2I). Notably, the GO term for the positive regulation of PCD (GO: 0043067) showed markedly lower gene enrichment in neonatal mice than in adults at all time points (Fig. 1K). This pattern of attenuated PCD enrichment was conserved in fully spinal cord-regenerating species, including zebrafish and salamanders (Fig. 1L and M). Consistent findings across species suggest that the precise regulation and maintenance of PCD homeostasis may contribute to their strong regenerative capacities.

Mitochondrial swelling, characterized by an increased mitochondrial diameter, is a key morphological marker of the intermediate-to-late stages of PCD [35,36]. Transmission electron microscopy (TEM) was used to examine the mitochondrial morphology in microglia, astrocytes, and neurons within the injury epicenter at 3 dpi in both adult and neonatal mice. Quantitative analysis revealed that adult mice had markedly larger mean mitochondrial diameters across all examined cell types than neonates, suggesting enhanced PCD activation in adults (Fig. 1N and O and Fig. S2J to M). To further validate these findings, we assessed DNA fragmentation using TUNEL (terminal deoxynucleotidyl transferase–mediated deoxyuridine triphosphate nick end labeling) staining [37]. Adult mice showed a 3.89-fold increase in TUNEL-positive cells at the injury site compared to neonates (Fig. 1P and Q). These findings suggest that moderate activation of PCD in neonatal mice may facilitate spinal cord regeneration.

### Thanatoset gene panel forms the fingerprint of pan-PCD post-SCI

**Fig. 2.**
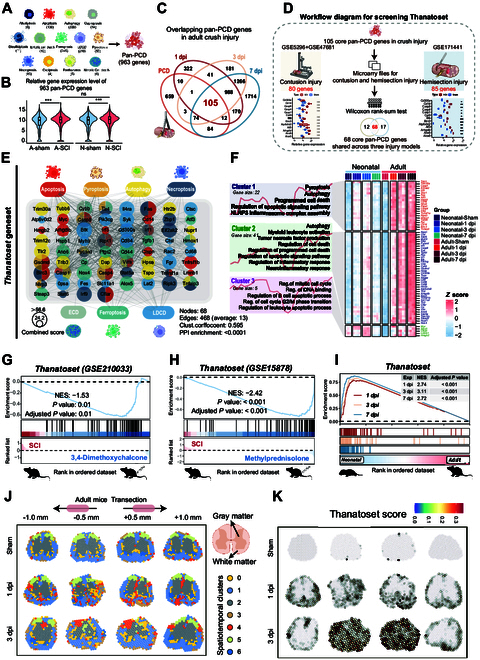
The Thanatoset gene panel identifies a pan-PCD gene signature post-SCI. (A) Total number of genes identified from 13 PCD single pathways collected from GSEA, WIKI, Reactome, Ferrdb, and PubMed databases. (B) Violin plot comparing the average expression levels of the 963 pan-PCD genes across different group in neonatal and adult mice post-SCI. (C) Venn diagram illustrating the overlap between DEGs in adult crush injury mice and the pan-PCD gene set. (D) Workflow for constructing the SCI-specific pan-PCD gene panel (Thanatoset), where 105 pan-PCD genes universally involved post-SCI were screened from crush injury models and further screened in contusion (80 genes, left panel) and hemisection (85 genes, right panel) models. (E) The Thanatoset encodes a highly interactive network comprising genes from 7 distinct PCD pathways. Node size represents the combined score with other molecules in the set, and gray lines indicate interactions. ECD, entotic cell death; LDCD, lysosome-dependent cell death. (F) Heatmap from Mufzz clustering analysis of genes in the Thanatoset with accompanying GO pathway enrichment analysis (*P* < 0.05). (G and H) GSEA results for the Thanatoset enrichment in adult SCI mice treated with 3,4-dimethoxychalcone (G) and methylprednisolone (H). (I) GSEA for the Thanatoset comparing adult versus neonatal mice post-SCI. (J) Spatial clustering of gene expression data from highly variable genes in the rostral and caudal regions (0.5 and 1 mm) of transverse sections in adult mice post-complete transection SCI. (K) AddModuleScore scoring of the Thanatoset in transverse sections of the spinal cord. Statistical analysis was conducted using the Wilcoxon rank-sum test (B and D). ns (not significant), *P < 0.05, **P < 0.01, ***P < 0.001.

The complex pathophysiology of PCD following SCI involves multiple subtypes with intricate interconnections and feedback mechanisms, thus posing a challenge in attributing overall cell death to any single pathway [9,10]. To address this, we conducted a comprehensive search across 4 databases (GSEA, WIKI, Reactome, and Ferrdb) as well as an extensive literature review [13,38–45], ultimately identifying 963 pan-PCD genes associated with 13 types of PCD (Fig. 2A and Table S1). Although both adult and neonatal mice showed a slight increase in the expression of these genes after crush injury (Fig. 2B), comparisons revealed no important overall differences in gene expression levels between the 2 groups post-SCI (Fig. 2B).

To establish an SCI-specific gene set for pan-PCD, we focused on DEGs during the critical early injury phases (1, 3, and 7 dpi) after crush SCI, identifying 105 pan-PCD genes actively involved in the injury response (Fig. 2C). To ensure robustness across different injury paradigms, we refined these 105 pan-PCD genes through rank-sum tests on microarray data from contusion (80 genes) and hemisection (85 genes) SCI models, which represent diffuse broader mechanical damage and partial nerve fiber transection, respectively (46, 47) (Fig. S3A and B). This rigorous approach yielded 68 core pan-PCD genes, designated as the “Thanatoset”, which demonstrated consistent involvement across various post-SCI phases and injury models (Fig. 2D and Table S2).

Seven major PCD types were identified within the 68 tightly interconnected genes in Thanatoset (protein–protein interaction, *P* < 0.0001) based on STRING analysis (Fig. 2E). Mufzz clustering further divided Thanatoset into 3 modules: cluster 1 (*Casp1, Zbp1, Ripk3*, etc.), cluster 2 (*Ctsl, Alox5, Cd84*, etc.), and cluster 3 (*Myc, Ripk1, Stat6*, etc.) (Fig. 2F). Cluster 1 exhibited marked enrichment in pyroptosis, autophagy, and apoptosis, whereas clusters 2 and 3 were also enriched in PCD-related pathways. Notably, cluster 2 was specifically associated with immune responses and neuroinflammation, whereas cluster 3 was linked to cell cycle regulation. These findings collectively validate the functional significance of PCD regulation and its critical role in SCI pathophysiology.

To evaluate the clinical relevance of Thanatoset, we reanalyze publicly available mRNA-seq data from drug-treated spinal cords post-injury. Treatment with 3,4-dimethoxychalcone, a compound known to modulate autophagy, pyroptosis, and necroptosis [46], markedly attenuated Thanatoset enrichment at 3 dpi (Fig. 2G). Similarly, methylprednisolone, a classical clinical agent with anti-inflammatory and anti-apoptotic properties [47], markedly reduced Thanatoset enrichment at 1 dpi, demonstrating the responsiveness of this gene panel to established PCD-modulating therapies (Fig. 2H). We then employed the Thanatoset gene panel to conduct comparative analyses of adult and neonatal post-SCI responses. Integrated GSEA analyses revealed markedly higher enrichment of Thanatoset genes in adult SCI mice across all injury phases (*P* < 0.001) (Fig. 2I). Within Thanatoset, *Gsdmd* and *Casp8* emerged as key regulators, exhibiting elevated mRNA and protein expression levels in injured adult mice compared to those in neonates (Fig. S3C). Immunofluorescence validation further confirmed the increased expression of GSDMD-N, a pyroptosis and inflammation mediator [48], and Cleaved-CASP8, a multifunctional mediator of apoptosis, necroptosis, and pyroptosis [49], in adults at 3 dpi (Fig. S3D and E). These findings suggest that the Thanatoset gene panel is a robust tool for investigating SCI-specific pan-PCD dynamics and effectively capturing the age-dependent differences in pan-PCD responses post-SCI.

### Spatial dynamics of pan-PCD responses post-SCI

To characterize the spatial dynamics and regional vulnerability of pan-PCD responses following SCI, we applied Thanatoset to spatial transcriptomics data (GSE256397) (50) from adult mouse spinal cords spanning an area 1 mm rostral and 1 mm caudal to the complete transection injury site (Fig. 2J). After quality control and dimensionality reduction, we identified 7 distinct spatial clusters that accurately delineated gray matter from white matter regions (Fig. 2J). Thanatoset scores progressively increased at 1 and 3 dpi, with pronounced enrichment observed in sections ±0.5 mm from the site at 3 dpi (Fig. 2K). Notably, white matter regions at the injury epicenter exhibited earlier Thanatoset enrichment (1 dpi), followed by subsequent propagation into the gray matter with greater activation at 3 dpi, highlighting the initial vulnerability of white matter and the temporal progression of the pathological cascade (Fig. 2K). These findings provide compelling evidence for the heightened sensitivity of white matter to pan-PCD in the context of SCI.

### Pronounced microglial pan-PCD in adult SCI

**Fig. 3.**
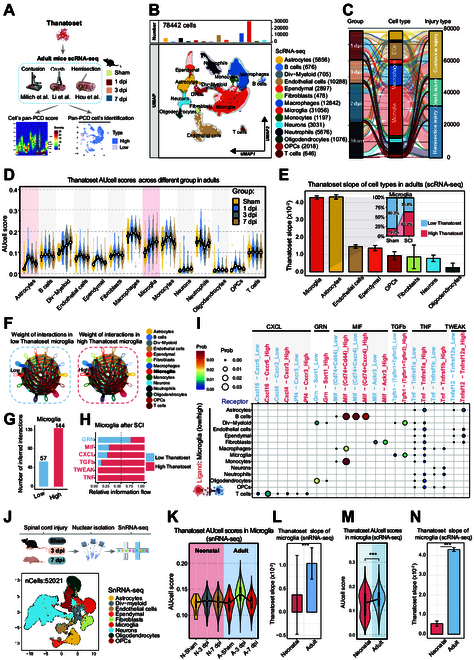
Microglial susceptibility to pan-PCD in adult mice post-SCI. (A) Workflow for identifying the pan-PCD response of cells across contusion, crush, and hemisection injury using the Thanatoset. (B) Uniform manifold approximation and projection (UMAP). visualization showing 14 distinct cell types identified across adult mouse contusion, crush, and hemisection SCI models in sham, 1 dpi, 3 dpi, and 7 dpi. (C) Sankey diagram illustrating the flow and distribution of each cell type across different experimental groups, encompassing 78,442 cells. (D) Thanatoset score comparison across different experimental groups (sham, 1 dpi, 3 dpi, and 7 dpi) for various cell types. (E) Slope of Thanatoset scores for different cell types post-SCI in adult mice relative to the sham group, modeled using a linear model [*model <- lm*(*AUC ~ Group*)]. The inset panel represents the proportions and counts of high versus low Thanatoset score cells in microglia pre- and post-SCI. (F) Cellular communication network in adult microglia (MG) with high and low Thanatoset scores and their interactions with all cell types post-SCI. (G) Interaction counts between adult microglia with high and low Thanatoset scores and all cell types post-SCI. (H) Relative pathway flow for key pathways in adult microglia with high versus low Thanatoset scores post-SCI. (I) Dot plot showing the interaction probability of high and low Thanatoset score microglia as ligands interacting with 14 receptor cell types via ligand–receptor pairs in adult mice post-SCI. Bar color and dot size both indicate cell interaction probability (Prob). (J) SnRNA-seq workflow and UMAP plot of spinal cord tissues from sham, 3 dpi, and 7 dpi mice at both adult and neonatal stages. (K) Violin plot comparing Thanatoset scores in microglia across different experimental groups in snRNA-seq. (L) Bar plot comparing the slope of Thanatoset scores for microglia between neonatal and adult mice post-SCI relative to sham groups in snRNA-seq. (M) Violin plot comparing Thanatoset scores in microglia across different ages post-SCI in snRNA-seq. (N) Bar plot comparing the slope of Thanatoset scores for microglia between neonatal and adult mice post-SCI relative to sham groups in scRNA-seq. Statistical analysis was conducted using the Wilcoxon rank-sum test (L, M, and N). ***P < 0.001.

In addition to its region-specific characteristics, PCD exhibits distinct cell-specific patterns within the injury microenvironment and may be influenced by the cell type, mechanical injury severity, and extent of the inflammatory response [8,51]. To delineate single-cell pan-PCD dynamics, we established a comprehensive single-cell RNA sequencing (scRNA-seq) reference atlas encompassing 3 SCI models in adult mice (crush, hemisection, and contusion) across multiple time points (sham, 1, 3, and 7 dpi) [51–53] (Fig. 3A). According to the standard scRNA-seq analysis pipeline, we identified 78,442 high-quality single cells from the spinal cord, which were classified into 14 distinct cell types, effectively capturing early cellular responses to SCI (Fig. 3B and C and Fig. S4A to G). By applying the Thanatoset gene panel, we generated a cellular pan-PCD atlas across cell clusters, revealing elevated Thanatoset AUCell scores in all cell types post-SCI (Fig. S5A to C). To validate the ability of Thanatoset to identify pan-PCD-sensitive cells, we utilized the automated threshold in AUCell software [area under the curve (AUC) > 0.13] to stratify cells into populations of low (46,431 cells) and high (32,011 cells) Thanatoset scores (Fig. S5D and E). GSEA results revealed that high-Thanatoset cells exhibited activation of cell death and inflammatory pathways, along with down-regulation of cellular maintenance pathways, confirming the effectiveness of Thanatoset for identifying pan-PCD-sensitive cell populations (Fig. S5F).

Given the baseline variability in Thanatoset scores across the 14 cell types under physiological conditions (Fig. 3D), we employed linear modeling to assess SCI-induced changes relative to the sham group, utilizing the slope of linear fit (Thanatoset slope) as a comparative metric for pan-PCD sensitivity (Fig. 3E). Among the resident cell types in the physiological spinal cord, microglia and astrocytes exhibited markedly steeper Thanatoset slopes than the other cell types (Fig. 3E), with microglia showing sustained increases in Thanatoset scores during 1 to 7 dpi (Fig. 3D). Notably, microglia exhibited the greatest proportional increase in high-Thanatoset cells, rising from 19.7% in the sham group to 63.2% post-SCI (Fig. 3E). These findings position microglia as the cell type most vulnerable to pan-PCD following SCI, characterized by the maximum Thanatoset slope and persistent post-injury activation.

Cells undergoing PCD can modulate the survival of neighboring cells through the release of damage-associated molecular patterns and soluble factors, thereby regulating intercellular communication [8,10]. CellChat analysis revealed markedly enhanced intercellular communication links in high-Thanatoset microglia compared to their low-scoring counterparts, highlighting their pivotal role in SCI-induced cellular interactions (Fig. 3F and G). Prominent differential interactions were identified in pathways including GRN, MIF, CXCL, TGF-β, TWEAK, and TNF signaling (Fig. 3H). High-Thanatoset microglia impaired adjacent healthy cells through up-regulated “CXCL”, “TNF”, and “TWEAK” signaling, ultimately hindering neural repair. Specifically, interactions between high-Thanatoset microglia and neurons, astrocytes, and oligodendrocytes were characterized by elevated TNF–TNFR ligand–receptor pairs (*Tnfrsf1a* and *Tnfrsf1b*), leading to cell death [54] (Fig. 3I). Conversely, GRN signaling, which confers anti-apoptotic effects to oligodendrocyte precursor cells (OPCs), was down-regulated in microglia with a high Thanatoset score [55] (Fig. 3I). These findings suggest that microglia with elevated pan-PCD activity exacerbate SCI pathology by amplifying inflammatory responses and suppressing reparative processes.

### Optimal post-SCI pan-PCD activation in neonatal microglia

We further leveraged a single-nuclei RNA sequencing (snRNA-seq) dataset (GSE234774), *Tabulae Paralytica*, which encompasses a comprehensive set of experimental conditions [56], to assess the universality of the Thanatoset gene panel for capturing pan-PCD responses (Fig. S5G). Consistent with the scRNA-seq data, Thanatoset scores were elevated across all cell types following SCI (1 to 7 dpi), except for low-abundance natural killer, B, and T cells (<0.5%), likely because of the lower sensitivity of snRNA-seq for immune cells [57] (Fig. S5G). Similarly, we assessed the Thanatoset slope across different cell types in the snRNA-seq dataset and found that astrocytes and microglia exhibited high Thanatoset slopes, with ependymal cells also showing notable elevation (Fig. S5H). For microglia at 7 dpi, we observed more pronounced Thanatoset scores in the microglia of aged mice, which exhibited poor recovery after SCI [58], than in those of adult mice (Fig. S5I and J). Additionally, male mice demonstrated higher Thanatoset scores than female mice, which is consistent with previous studies reporting heightened inflammatory responses in male microglia after SCI [59] (Fig. S5I and J). These findings further validate the effectiveness of the Thanatoset gene panel as a tool for identifying pan-PCD responses in microglia after SCI via snRNA-seq.

To further investigate the dynamics of microglial pan-PCD in neonatal and adult mice following SCI, we conducted snRNA-seq on spinal cord tissues harvested from sham, 3-dpi, and 7-dpi mice at both adult and neonatal stages (Fig. 3J). Among the 9 identified cell subsets, neurons in neonatal mice experienced a slower decline in the acute phase of injury compared to adult mice, suggesting a more favorable regenerative environment in neonatal mice (Fig. S6A). We then assessed these nuclei using the Thanatoset gene panel and found that multiple functional cell types (microglia, ependymal cells, OPCs, and neurons) exhibited steeper Thanatoset slopes in adult mice than in neonatal mice (Fig. S6B and C). Specifically, in microglia, Thanatoset scores post-SCI were markedly higher in adult mice, whereas those in neonatal mice were similar to the scores for their physiological state with a more gradual slope (Fig. 3K and L).

Given that scRNA-seq offers better sensitivity for capturing microglial cells than snRNA-seq, we used the label transfer function of *Symphony* to annotate neonatal SCI scRNA-seq data (GSE150871) (sham, 3 dpi, and 5 dpi) and further evaluate age-dependent differences in microglial pan-PCD [28] (Fig. S6D to F). Adult microglia (21,476 cells) exhibited markedly higher Thanatoset scores post-SCI than neonatal microglia (13,795 cells) (Fig. 3M and Fig. S6G). The post-injury Thanatoset slope of adult microglia was 7.94 times steeper than that observed in neonatal microglia, suggesting that adult microglia are more susceptible to pan-PCD following SCI (Fig. 3N). Additionally, costaining of injured tissues at 3 dpi with the lysosome-dependent cell death (LDCD) marker CTSL and microglial marker Tmem119 revealed that the colocalization ratio of *Tmem119^+^Ctsl^+^* cells in adult mice was 1.46 times higher than that in neonatal mice (Fig. S6H and I). These findings reveal that the post-SCI pan-PCD response of microglia is markedly stronger in adult mice than in neonatal mice.

### Virtual screening identifies potential pan-PCD-modulating compounds

**Fig. 4.**
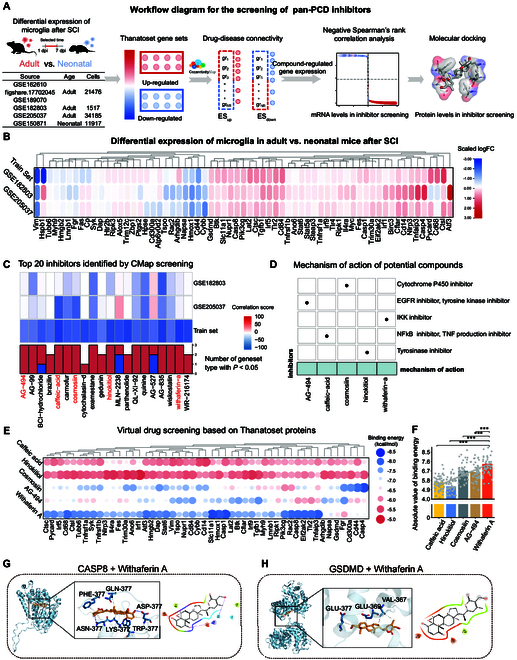
Screening of small-molecule inhibitors targeting pan-PCD in microglia after adult SCI. (A) Workflow diagram illustrating the screening process for pan-PCD inhibitors. (B) Heatmap showing the differential expression of Thanatoset genes in microglia from adult and neonatal mice post-SCI. Scaled logFC values are presented. The training set includes all SCI microglia from Fig. 3B (21,476 cells), and validation sets include GSE182803 (1,517 cells) and GSE205037 (34,185 cells). (C) Top 20 inhibitors identified by CMap screening across datasets, ranked by negative correlation score (upper panel). In the lower panel, red indicates the number of datasets with markedly up-regulated Thanatoset genes, while blue indicates the number of datasets with markedly down-regulated Thanatoset genes (*P* < 0.05). (D) MOA of the selected 5 inhibitors identified through CMap analysis. Dot size represents the number of inhibitors within each functional category. (E) Heatmap of molecular docking binding energy for the selected 5 inhibitors with 49 Thanatoset proteins. Bubble size and color represents binding energy (kcal/mol). (F) Bar graph comparing the average absolute binding energy (kcal/mol) of candidate small molecules with Thanatoset proteins. (G and H) Molecular docking visualization of withaferin A binding to key Thanatoset proteins: CASPASE-8 (G) and GSDMD (H). Statistical analysis was conducted using one-way ANOVA with Tukey (F). ***P < 0.001.

To reprogram adult SCI microglial pan-PCD toward a neonatal-like state, we employed the Connectivity Map (CMap) database to identify compounds capable of reversing pan-PCD phenotypes through virtual drug screening in microglia post-SCI (Fig. 4A). To enhance the robustness of our predictions, we expanded our analysis to 21,476 microglia from adult mice after SCI (Fig. 3B) by incorporating 2 additional datasets, GSE182803 (1,517 microglial cells) [60] and GSE182087 (34,185 microglial cells) [58], improving the reliability of the dataset for drug prediction (Fig. S7A and B). Differential expression analyses were conducted between adult and neonatal microglia post-SCI (11,917 cells) using the logFC (fold change) values of Thanatoset genes as input gene signatures for drug prediction (Fig. 4B). The CMap database identified 20 compounds that were negatively correlated with the Thanatoset gene signature in adults, indicating their potential to reverse pan-PCD expression profiles in adult microglia (Fig. 4C). Among these, 12 compounds (*P* < 0.05) consistently exhibited negative regulation across all 3 datasets (Fig. 4C, bottom panel). A literature review highlighted 5 compounds with favorable biosafety profiles: AG-494 [61], caffeic acid [62], cosmosiin [63], hinokitiol [64], and WFA [65]. Mechanism-of-action (MOA) analyses further revealed that WFA and caffeic acid function as NF-κB inhibitors, *AG-494* and hinokitiol function act as tyrosine kinase inhibitors, and cosmosiin functions as a cytochrome P450 inhibitor (Fig. 4D).

Predicting small-molecule regulation of pan-PCD based solely on transcriptomic dynamics provides limited insights into protein functions, conformational changes, and active states. To address this limitation, we conducted a molecular docking simulation for the 5 candidate compounds against 49 fully resolved mouse proteins from Thanatoset (Fig. 4E). Among these, WFA exhibited the lowest average binding energy (–7.36 ± 0.97 kcal/mol), indicating the strongest binding affinity (Fig. 4F). To characterize the structural basis of these interactions, we mapped the key binding sites and pockets of major Thanatoset proteins, including GSDMD, CASP8, CTSL, and CD68 (Fig. 4G and H and Fig. S7C and D). Additionally, WFA exhibited a molecular weight of 426.6 Da and a logarithm of the partition coefficient (octanol/water) (Log Po/w) of 3.42, satisfying the criteria for favorable blood–brain barrier permeability, thereby highlighting its ability to effectively target SCI sites (Table S3). These findings establish WFA as a promising candidate for reprogramming microglial pan-PCD in adult mice, potentially enabling the transition to a neonatal-like state following SCI.

### WFA alleviates microglial pan-PCD in vitro

**Fig. 5.**
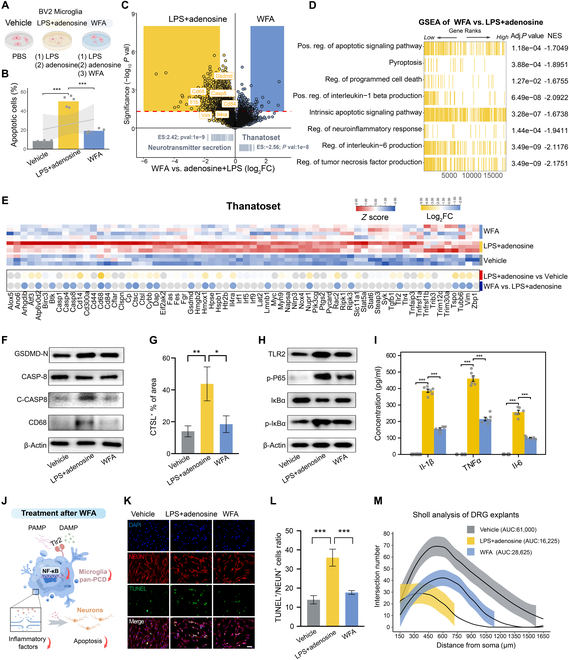
WFA attenuates microglial pan-PCD and promotes neuroprotection in vitro. (A) Schematic representation of BV2 microglial treatments: vehicle group (PBS treatment), LPS + adenosine group (LPS followed by adenosine treatment), and WFA group (LPS + adenosine followed by WFA intervention). (B) Quantification of apoptotic microglial cells (B2 + B4) (%) via flow cytometry. (C) Volcano plot of gene expression differences between WFA-treated and LPS + adenosine-treated groups. Specific GSEA pathways (Neurotransmitter secretion and Thanatoset) enriched in each group are displayed below. (D) GSEA enrichment comparing WFA-treated microglia to LPS + adenosine-treated microglia. (E) Heatmap of Thanatoset gene expression across vehicle, LPS + adenosine, and WFA groups. The upper panel shows *Z* scores (scaled FPKM values), while the lower dot plot highlights genes with log_2_|FC| > 1 and *P* < 0.05, where yellow represents up-regulated genes and blue represents down-regulated genes. (F) Western blot analysis of GSDMD-N, CASP8, Cleaved-CASP8, and CD68 protein levels across groups, demonstrating reduced protein levels in WFA-treated groups compared to LPS + adenosine groups. (G) Quantification of CTSL^+^ microglial area across groups (*n* = 3). (H) Western blot analysis of NF-κB pathway components (TLR2, p-P65, IκBα, and p-IκBα). (I) ELISA analysis of inflammatory cytokines (IL-1β, TNF-α, and IL-6) in culture supernatants (*n* = 5). (J) Schematic of the Transwell coculture system used to assess the effects of WFA-treated microglia on neurons. (K) Representative immunofluorescence images of neurons cocultured with microglia, showing NEUN and TUNEL costaining across groups. (L) Quantification of TUNEL^+^ and NeuN^+^ colocalization ratios (*n* = 3). (M) Sholl analysis of DRG neuron explants cocultured with 3 different microglial groups. Statistical analysis was performed using one-way ANOVA with Tukey (B, G, I, and L). *P < 0.05, **P < 0.01, ***P < 0.001.

To investigate the ability of WFA to attenuate pan-PCD in microglia, we established an in vitro model that mimics the pan-PCD observed in microglia post-SCI. As microglial pan-PCD is triggered by dual stimulation from damage-associated molecular patterns and pathogen-associated molecular patterns [66], BV2 microglial cells were treated sequentially with lipopolysaccharide (LPS) for 24 h, followed by adenosine for 1 h [67] (Fig. 5A). Flow cytometric analyses revealed that LPS + adenosine treatment significantly increased the percentage of apoptotic cells (B2 + B4) from 8.54% to 50.20%, confirming the induction of microglial pan-PCD (Fig. 5B). To evaluate the therapeutic potential of WFA in rescuing microglia from pan-PCD, we treated LPS + adenosine-treated microglia with WFA at concentrations of 0 to 500 nM. Cell viability assays revealed that WFA concentrations ≥300 nM effectively enhanced microglial survival (Fig. S8A). Furthermore, 300 nM WFA reduced the proportion of apoptotic cells by 30.96%, demonstrating its ability to mitigate microglial apoptosis and suggesting its potential therapeutic role in alleviating pan-PCD (Fig. 5B and Fig. S8B to D).

To further investigate the effects of WFA on microglial pan-PCD, transcriptome sequencing was performed on microglia from 3 treatment groups: phosphate-buffered saline (PBS), LPS + adenosine, and WFA (Fig. S8E). The Thanatoset gene set was markedly enriched in LPS + adenosine-treated microglia (*P* < 0.001) (Fig. S8F), but markdely down-regulated in WFA-treated microglia (*P* < 0.001) (Fig. 5C). Similarly, GSEA analysis revealed that WFA suppressed the pathways associated with pyroptosis, apoptosis, and neuroinflammatory responses, further demonstrating its potential to ameliorate the injury microenvironment (Fig. 5D). A heatmap analysis revealed that 72.05% of Thanatoset genes (49 genes) that were up-regulated by LPS + adenosine treatment were markedly down-regulated following WFA treatment (*P* < 0.05) (Fig. 5E). Among these, several key Thanatoset genes, such as *Gsdmd*, *Casp8*, *Cd68*, and *Ctsl*, were markedly down-regulated in WFA-treated microglia. Western blotting confirmed these findings, showing reduced protein levels of GSDMD-N, Cleaved CASPASE-8, and CD68 (Fig. 5F and Fig. S8G). Immunofluorescence analysis further validated the decrease in CTSL-positive areas following WFA treatment (Fig. 5G and Fig. S8I). Collectively, these results demonstrate that WFA effectively attenuates the pan-PCD profile in microglia in vitro*.*

Next, we sought to elucidate the upstream signaling pathways involved in the regulation of pan-PCD in microglia. Given that both LPS and adenosine activate the NF-κB signaling pathway, we performed GSEA analysis and identified substantial enrichment of NF-κB signaling in LPS + adenosine-treated microglia (*P* < 0.001) (Fig. S8J). Notably, WFA treatment markedly down-regulated NF-κB signaling in these microglia, thereby mitigating pan-PCD (Fig. S8K). Western blot analyses confirmed that WFA markedly reduced Toll-like receptor 2 (TLR2) expression and the phosphorylation of NF-κB p65 (p-P65) and IκBα (p-IκBα), indicating attenuation of NF-κB activation (Fig. 5H and Fig. S8H). Furthermore, enzyme-linked immunosorbent assays (ELISAs) demonstrated that WFA suppressed the release of key NF-κB downstream inflammatory cytokines, including TNF-α, interleukin-1β (IL-1β), and IL-6 (30) (Fig. 5I).

Given the critical role of microglia-derived inflammatory mediators in neuronal damage post-SCI, we employed a Transwell coculture system to assess the effects of WFA-treated microglia on VSC4.1 neuron-like cells (Fig. 5J). VSC4.1 cells cocultured with WFA-treated microglia exhibited markedly fewer TUNEL-positive cells than those cocultured with LPS + adenosine-treated microglia, indicating enhanced neuronal survival (Fig. 5K and L). Additionally, WFA-treated microglia increased the release of neurotrophic factors such as nerve growth factor and brain-derived neurotrophic factor, further supporting their neuroprotective role (Fig. S8L). To evaluate axonal outgrowth, we directly cocultured microglia from the PBS, LPS + adenosine, and WFA groups with dorsal root ganglion (DRG) explants and neurons. The average length of Tuj1^+^ neurites in the WFA-treated group (65.90 ± 2.82 μm) was markedly longer than that in the LPS + adenosine group (42.91 ± 12.11 μm), further highlighting the neuroprotective effects of WFA (Fig. S8M and N). Sholl analysis revealed that the AUC for branching complexity in DRG explants was approximately 1.76-fold higher in the WFA-treated group than in the LPS + adenosine group (Fig. 5M and Fig. S8O). These findings demonstrate that WFA effectively reduces pan-PCD in microglia, promoting neuroprotection, and highlight its potential as a therapeutic agent for enhancing neuronal survival and axonal repair in vivo.

### WFA enhances post-SCI functional recovery in adults

**Fig. 6.**
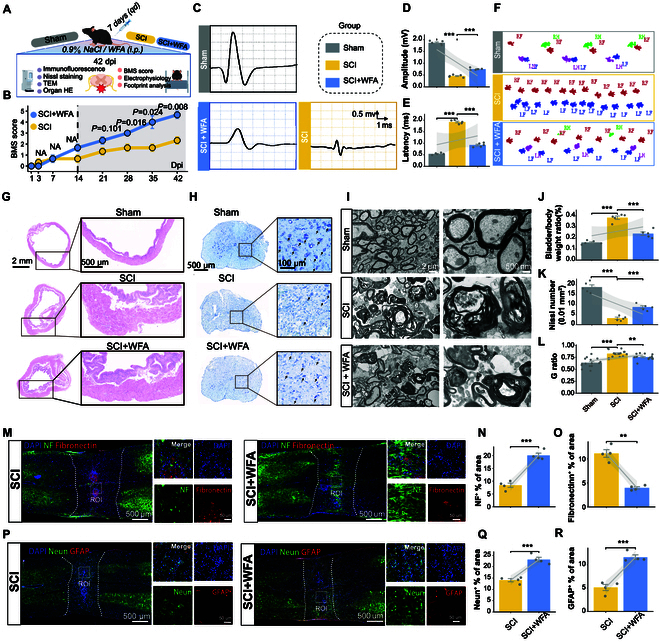
WFA promotes long-term functional recovery post-SCI in adult mice. (A) Schematic diagram illustrating experimental groups: sham, SCI group, and SCI + WFA group. (B) Line graph comparing BMS scores between SCI and SCI + WFA groups over 42 dpi. (C) Representative MEP waveforms across sham, SCI, and SCI + WFA groups. (D and E) Quantification of MEP amplitudes (mV) (D) and latencies (ms) (E) across groups (*n* = 5). (F) Representative gait trajectories from CatWalk analysis: RF, right forelimb; LF, left forelimb; RH, right hindlimb; LH, left hindlimb. (G to I) Representative histological images of tissues at 42 dpi. (G) H&E staining of bladder tissues. (H) Nissl staining of injury regions. (I) TEM images of myelin structures. (J) Quantification of bladder weight normalized to body weight (*n* = 5). (K) Quantification of Nissl-stained motor neurons in the spinal cord ventral horn (*n* = 5). (L) Quantification of G ratio (axon diameter to fiber diameter) measured from TEM images (*n* = 15). (M) Representative images of NF200 and fibronectin staining in the lesion core at 42 dpi. (N and O) Quantification of NF200^+^ area (N) and fibronectin^+^ area (O) per spinal cord section (*n* = 5). (P) Representative images of NeuN and GFAP staining in the lesion core at 42 dpi. (Q and R) Quantification of NeuN^+^ area (Q) and GFAP^+^ area (R) per spinal cord section (*n* = 5). Statistical analysis was conducted using 2-tailed *t* tests (N, O, Q, and R) or one-way ANOVA with Tukey (D, E, and J to L). **P < 0.01, ***P < 0.001.

To evaluate the therapeutic effects of WFA on functional recovery, we established a crush SCI model in adult mice and administered WFA (dose: 5 mg/kg) via intraperitoneal injection for 7 consecutive days post-SCI (Fig. 6A). Functional recovery was assessed using the Basso Mouse Scale (BMS) score, which showed marked improvement in WFA-treated mice at 28 dpi (*P* = 0.016), 35 dpi (*P* = 0.024), and 42 dpi (*P* = 0.008) compared with sham-operated mice (Fig. 6B). Electrophysiological monitoring of motor-evoked potentials (MEPs) revealed a 64% increase in amplitude and a 52% reduction in latency in the WFA-treated group, suggesting enhanced neural conductivity (Fig. 6C to E). Additionally, CatWalk gait analysis indicated that WFA-treated mice exhibited more coordinated gait patterns and stronger plantar pressure, signifying improved motor coordination and function (Fig. 6F and Fig. S9A and B). Furthermore, pathological examinations of bladder tissues, a common site of SCI-related complications, revealed that WFA treatment reduced the relative bladder weight by 38% and alleviated mucosal edema and muscle bundle disorganization, suggesting the recovery of autonomic nervous function in WFA-treated SCI mice (Fig. 6G and J).

At the tissue level, Nissl staining at 42 dpi demonstrated a 151% increase in the number of motor neurons in the ventral horn of the spinal cord in WFA-treated animals (Fig. 6H and K). TEM analysis of the injury core further demonstrated enhanced myelination, as evidenced by preservation of myelin structures and a slight reduction in the G ratio (axon diameter to fiber diameter) (Fig. 6I and L). Concurrently, immunofluorescence staining of the injured area corroborated these findings, showing increased NF200 fluorescence and reduced fibronectin fluorescence in WFA-treated SCI mice, indicating enhanced neuronal regeneration and reduced scar formation following WFA treatment (Fig. 6M to O). Moreover, the lesion core of the WFA-treated group exhibited a 64% increase in the NeuN^+^ area and a 126% increase in the GFAP^+^ area (Fig. 6P to R). These morphological improvements were strongly correlated with the observed enhancement in neurological recovery. Notably, no pathological abnormalities were detected in the major organs (heart, liver, spleen, lungs, and kidneys) of WFA-treated mice, confirming its biosafety (Fig. S9C).

### WFA modulates the injury microenvironment by reducing microglial pan-PCD

**Fig. 7.**
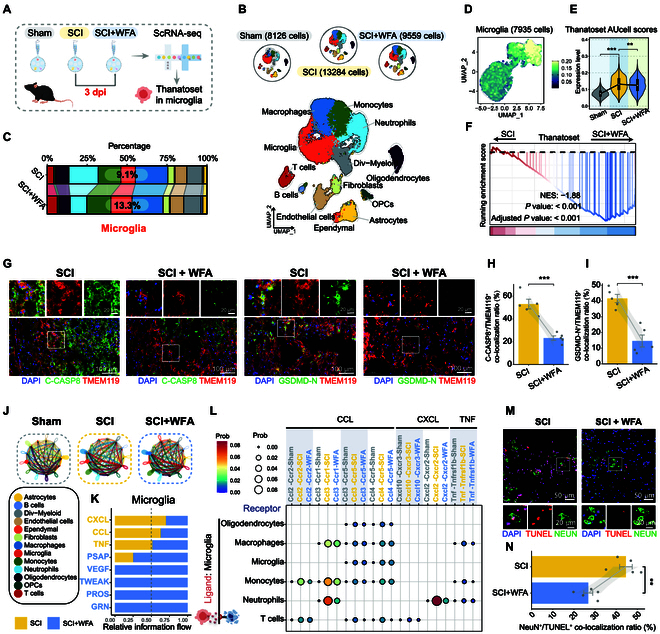
WFA attenuates microglial pan-PCD and protects cells in the injury microenvironment post-SCI. (A) Schematic overview of the scRNA-seq analysis of spinal cord samples collected at 3 dpi from sham, SCI, and SCI + WFA mice. (B) UMAP visualization of 30,969 cells categorized into 13 cell types across sham, SCI, and SCI + WFA groups. (C) Stacked bar graph showing the proportion of cell types across sham, SCI, and SCI + WFA groups. (D) UMAP visualization of 7,935 microglia cells for pan-PCD assessment using the Thanatoset gene panel. (E) Violin plots comparing Thanatoset scores in microglia across the 3 groups. (F) GSEA plot comparing the Thanatoset gene set between the SCI + WFA group and the SCI group. (G) Representative immunofluorescence images showing costaining of TMEM119 (microglial marker) with pan-PCD markers C-CASP8 and GSDMD-N. (H and I) Quantification of C-CASP8^+^/TMEM119^+^ (H) and GSDMD-N^+^/TMEM119^+^ (I) cell colocalization ratios (*n* = 5). (J) Cell–cell communication networks of 13 cell types across sham, SCI, and SCI + WFA groups. (K) Relative information flow of key microglial signaling pathways. (L) Dot plot showing specific ligand–receptor interactions between microglia (as ligands) and all cell types (as receptors). Bar color and dot size indicate cell interaction probability. (M) Representative immunofluorescence images of NeuN^+^/TUNEL^+^ neurons in the injury region. (N) Quantification of NeuN^+^/TUNEL^+^ cell colocalization ratios (*n* = 5). Statistical analysis was conducted using 2-tailed *t* tests (H, I, and N) or one-way ANOVA with Tukey (E). **P < 0.01, ***P < 0.001.

To investigate the effect of WFA on acute phase microglial pan-PCD reduction post-SCI, we conducted scRNA-seq analysis on spinal cord tissues from sham-operated (sham), crush-injured (SCI), and WFA-treated crush-injured (SCI + WFA) mice at 3 dpi (Fig. 7A). After quality control, we identified 30,969 cells, which were categorized into 13 distinct cell types (Fig. 7B and Fig. S10A). Notably, WFA treatment resulted in a 4.2% increase in the relative proportion of microglia following SCI (Fig. 7C). We then isolated 7,935 microglia and assessed their pan-PCD response using the Thanatoset gene panel and observed that WFA markedly reduced the elevated pan-PCD levels in microglia post-SCI [normalized enrichment score (NES) = −1.88, *P* < 0.001] (Fig. 7D to F and Fig. S10B and C). Consistent with our in vitro findings, WFA suppressed inflammatory responses and inhibited NF-κB signaling in microglia following SCI (Fig. S10D). Immunofluorescence costaining verified that WFA treatment markedly reduced colocalization of the microglial marker TMEM119 and key Thanatoset proteins Cleaved-CASP8 and GSDMD-N in the injured region after WFA treatment (Fig. 7G to I). These results indicate that WFA effectively alleviates microglial pan-PCD during the subacute phase of SCI.

To further examine the effects of WFA-mediated microglial pan-PCD reduction on the SCI microenvironment, we generated cell–cell communication networks for the sham, SCI, and SCI + WFA groups (Fig. 7J). WFA treatment exhibited dual regulatory effects, suppressing pro-apoptotic signaling pathways (CXCL, CCL, and TNF) while enhancing neuroprotective pathways (PASP, VEGF, TWEAK, PROS, and GRN) (Fig. 7K).

Specifically, WFA-treated microglia exhibited attenuated CCL signaling to oligodendrocytes and immune cells (neutrophils, monocytes, and macrophages), decreased CXCL signaling to neutrophils and T cells, and diminished TNF signaling in monocytes and macrophages (Fig. 7L). These changes suggest that WFA modulates microglia-mediated inflammatory responses by suppressing immune cell activation and reducing oligodendrocyte damage. Simultaneously, WFA enhanced beneficial signaling pathways, including GRN-mediated anti-inflammatory signaling between microglia and oligodendrocytes, PASP-mediated neuroprotection in ependymal cells and OPCs, and vascular endothelial growth factor (VEGF)-induced angiogenesis in endothelial cells (Fig. S10E). As scRNA-seq does not capture neurons, we performed NeuN^+^/TUNEL^+^ immunofluorescence staining to evaluate the effects of WFA on neuronal apoptosis (Fig. 7M). Quantification revealed that WFA treatment resulted in a 40% reduction in neuronal apoptosis within the injury region (Fig. 7N). These findings provide compelling evidence that the WFA-mediated attenuation of microglial pan-PCD protects cells within the injury microenvironment during the early stages of SCI.

## Discussion

In adult mammalian SCI, excessive activation of PCD drives pathological processes, including inflammation, oxidative stress, demyelination, and impaired axonal regeneration, ultimately leading to persistent dysfunction [8]. Here, we demonstrated that microglia in neonatal mice exhibit an “optimal” pan-PCD response post-SCI, which serves as an ideal state for adult rodents to emulate. We also identified WFA as a potential therapeutic agent capable of reprograming microglial pan-PCD in adult rodents to a neonatal-like state, ultimately promoting spinal cord repair.

Previous studies have shown that neonatal mice exhibit remarkable regenerative abilities in organs such as the heart and skin shortly after birth [24,25]. Comparative analyses of biological processes in neonatal and adult mice have provided valuable insights into enhancing regenerative capacity in adults. For example, mechanisms such as SphK2 activation in cardiomyocytes and Lef1 expression in fibroblasts markedly enhance regenerative potential and have been successfully validated in adult mouse models [24,25]. Similarly, the present study reveals that neonatal mice exhibit characteristics of highly regenerative species, such as adult axolotls and zebrafish, where appropriately activated PCD following SCI primarily functions to clear necrotic cells and debris, creating a conductive environment for tissue repair and growth [19,29]. This key pathological signature in neonatal mice could serve as a model for adult mammals, providing a potential pathway to enhance regenerative capacity in both adult rodents and potentially humans post-SCI.

Pan-PCD, a unified framework integrating diverse PCD pathways, offers insights into advancing disease understanding and treatment by elucidating the interplay between apoptosis, necroptosis, pyroptosis, ferroptosis, autophagy, and other emerging modalities [11,68]. Pan-PCD composition varies across different diseases and tissues; in the present study, we identified 5 well-known PCD types and 2 less-explored types [LDCD and entotic cell death (ECD)], forming an SCI-specific pan-PCD gene set. In cancer biology, cancer-specific pan-PCD has been shown to modulate tumor immunity, therapy resistance, and metastasis, with emerging evidence suggesting that the pan-PCD framework may enhance immune checkpoint inhibitor therapies [13]. Similarly, disease-specific pan-PCD serves as a transformative paradigm bridging molecular biology and clinical practice, with particular applications in neurodegeneration and inflammation [12,68]. Our findings indicate that pan-PCD influences cell survival, modulates neuroinflammatory responses, and regulates growth factor secretion, ultimately impacting SCI prognosis. By providing a critical framework for simulating neonatal-like regenerative environments in adult SCI, the pan-PCD framework accelerates drug discovery to promote functional recovery.

In the injury area of adult SCI, neuronal cell bodies and glial cells undergo irreversible necrosis within hours owing to mechanical damage, triggering inflammatory responses and oxidative stress, which subsequently induce PCD in the injury penumbra [69]. The present study revealed that the white matter of the injury penumbra is particularly vulnerable to early pan-PCD during the acute phase (1 to 7 dpi) following SCI. Among white matter cells, oligodendrocytes exhibited a significant increase in pan-PCD at 1 dpi, which declined by 3 to 7 dpi. Similarly, OPCs, astrocytes, endothelial cells, and fibroblasts, which are key components of the white matter and blood–brain barrier, followed a similar trend. This early vulnerability is likely due to direct contact of white matter axons and the blood–brain barrier with the core injury site, exposing them to inflammation, excitotoxicity, and oxidative stress spreading from the injury core [8]. In contrast, neurons in the gray matter of the core injury area did not display an early increase in pan-PCD but exhibited a sustained rise over time. This delayed response may be due to the larger size of the neurons and longer time required for the pan-PCD to develop fully [56]. Among resident glial cells, microglia post-SCI in adults exhibited the most pronounced increase in pan-PCD during the acute phase, likely driven by inflammatory stress, oxidative burden, and the requirement to clear necrotic cells and debris [70]. These findings highlight the differential sensitivity of various cell types to pan-PCD after SCI and reveal distinct temporal and spatial vulnerability patterns.

Our results show that, compared to the excessive activation of pan-PCD in microglia following SCI in adult mice, neonatal mice maintain a pan-PCD state that is essentially consistent with their physiological condition, as observed in both SCI-induced scRNA-seq and snRNA-seq data. This may be a key reason why neonatal mice maintain higher neuronal survival than adult mice during the acute phase of injury. In recent years, the CMap framework has been extensively used to identify potential therapeutic targets and pathways by analyzing gene sets, predicting regulatory effects on cellular behavior, and improving treatment strategies for conditions such as abdominal aortic aneurysms and obesity [71,72]. Our comprehensive drug prediction analyses identified WFA as a potent regulator of microglial pan-PCD states in adult SCI, simulating neonatal-like regenerative patterns. WFA, a natural steroidal lactone, has previously been reported to modulate oxidative stress and inflammatory responses in microglia during brain injury [73]. The present study further demonstrated that WFA markedly reduced microglial pan-PCD levels and protected neurons by modulating the NF-κB signaling pathway to decrease the secretion of inflammatory factors. These findings suggest that integrating transcriptomic signatures with protein structure-based predictions provides a robust pipeline for identifying small molecules capable of reversing pan-PCD and promoting tissue regeneration.

Despite these promising findings, our study has certain limitations. First, although we included 13 types of PCD, ongoing research is likely to uncover additional forms. Building a real-time traceable database of PCD genes and types with continuous updates represents a valuable future research direction. Second, although the Thanatoset gene panel effectively captured SCI-specific pan-PCD, the underlying molecular interactions and complex regulatory networks require further investigation through in vitro and in vivo studies. Lastly, although WFA shows promise in promoting spinal cord repair, its known pleiotropic effects, such as cytoskeletal modulation and heat shock protein induction, may present off-target risks, particularly due to the lack of microglia-specific targeting in our model. To improve the translational potential of WFA for human SCI treatment, developing targeted delivery systems, such as nanoparticle-based platforms, is essential to enhance therapeutic precision and minimize off-target interactions [74].

In conclusion, uncontrolled PCD induced by adult SCI triggers complex and overlapping pathological events, leading to extensive tissue and cellular damage. Therefore, we developed the Thanatoset gene set to accurately capture pan-PCD responses at both tissue and cellular levels during the acute phase of SCI. Furthermore, by integrating computational and experimental approaches, we identified WFA as a promising therapeutic agent capable of modulating microglial pan-PCD in adult SCI to mimic neonatal patterns, thereby promoting spinal cord repair. These findings establish a framework for utilizing neonatal PCD dynamics to enhance tissue repair in various adult tissue injury contexts.

## Methods

### Mouse model and ethics statement

All animal procedures and surgeries were conducted with approval from the Ethical Committee for Animal Experiments of the Fourth Military Medical University (License No. IACUC-20240363). All mice were housed in normal experimental cages on a 12-h/12-h light/dark cycle and provided access to food and water ad libitum. Newborn C57BL/6 pups (P2) were anesthetized with isoflurane, while adult female C57BL/6 mice (8 to 10 weeks old, weighing 18 to 20 g) were anesthetized via intraperitoneal injection of a combination of ketamine (87.5 mg/kg) and xylazine (12.5 mg/kg).

Spinal cord crush injuries were performed as previously described [28,53]. Briefly, a laminectomy was carried out at the thoracic level (T9–T10) to fully expose the spinal cord from side to side. The spinal cord was then crushed for 2 s using No. 5 Dumont forceps (Fine Science Tools) with a 0.1-mm tip width at the last 5 mm of the forceps. In the sham group, only the laminectomy was performed without subsequent SCI. Adult mice in the WFA group were administered WFA (Sigma-Aldrich, #681535) via intraperitoneal injection at 5 mg/kg daily for 7 dpi. The SCI group received an equal volume of PBS as a control. Postoperative care included the application of diclofenac sodium cream for pain relief, and the animals were kept warm overnight using a heating pad. Pups were returned to their mother’s cage after covering them with maternal feces. Manual bladder expression was performed daily until the mice were euthanized.

### Behavioral assessments

The BMS was used to assess hind-limb movement in adult mice, utilizing a 9-point scale in an open-field environment. Mice (*n* = 5 per group) were evaluated both before and after injury by the same 3 observers, who were blinded to the experimental conditions to ensure a double-blinded assessment. Gait analysis was performed using the CatWalk XT system (Noldus, Netherlands) for both neonatal and adult mice. CatWalk software (v10.6) was used to mark pawprints and analyze gait parameters during locomotion.

### Electrophysiological analysis

At 42 dpi, electrophysiological tests were performed to assess the functional status of sensorimotor signal conduction (*n* = 5 per group). Under anesthesia, the sciatic nerves of the mice were exposed. For MEP measurements, a stimulating electrode was placed on the spinal motor cortex, while a recording electrode was positioned on the sciatic nerve. The waveforms, amplitudes, and latencies of the MEPs were recorded and subsequently analyzed.

### Tissue section preparation and H&E and Nissl staining

Mice from the sham group, as well as those at 3 and 42 dpi, were euthanized with a lethal dose of anesthesia and transcardially perfused with PBS, followed by 4% paraformaldehyde (PFA). After fixation, the spinal cord and major organs were collected. The tissues were incubated in a 30% sucrose solution at 4 °C for cryoprotection. Transverse and sagittal sections were cut at 10-μm thickness using a cryostat and stored at −20 °C until further processing. Sections were stained with standard hematoxylin and eosin (H&E) pipeline. For Nissl staining, spinal cord sections were placed on a humidified tray and covered with Nissl staining solution (Servicebio). Bright-field images were acquired using a light microscope (Olympus, Japan).

### Immunohistochemistry

Prior to staining, sections were air-dried at room temperature for 30 min and then re-immersed in 4% PFA for 10 min. To block nonspecific antibody binding and enhance cell membrane permeability, sections were incubated in a solution containing 0.3% Triton X-100 and 1% goat serum at room temperature for 1 h. The following primary antibodies were used: rabbit anti-NF200 (#N4142, Sigma-Aldrich, 1:200), chicken anti-GFAP (ab4674, Abcam, 1:500), rabbit anti-GSDMD-N (#DF13758, Affinity, 1:100), rabbit anti-Cleaved-CASP8 (#8592, Cell Signaling Technology, 1:200), rabbit anti-CTSL (orb539069, Biorbyt, 1:50), mouse anti-TMEM119 (#98778, Cell Signaling Technology, 1:100), rat anti-NEUN (ab279297, Abcam, 1:200), and rabbit anti-fibronectin (ab2413, Abcam, 1:100).

Cells or DRG tissue blocks were fixed with 4% PFA for 30 min, permeabilized with 0.3% Triton X-100 for 10 min, and blocked with 1% goat serum for 1 h at room temperature. Samples were incubated overnight at 4 °C with primary antibodies: rabbit anti-CTSL (Biorbyt, #orb539069, 1:50), rabbit anti-NEUN (Abcam, ab177487, 1:200), mouse anti-βIII tubulin (TUJ1, Abcam, #ab78078, 1:200), and rabbit anti-S100 beta (#ab52642, Abcam, 1:200). After washing with PBS, samples were incubated with fluorophore-conjugated secondary antibodies for 1 h at room temperature in the dark.

To quantify cell apoptosis, TUNEL staining was performed using the TMR (red) TUNEL Cell Apoptosis Detection Kit (Servicebio, G1502-50T) according to the manufacturer’s instructions. After incubation with 4′,6-diamidino-2-phenylindole (DAPI) working solution for 10 min in the dark at room temperature, sections were washed with PBS. Imaging was performed using a laser scanning confocal microscope (LSCM; NIKON). Image analysis was performed using Fiji software (v2.1.0).

### TEM analysis

Spinal cord tissue was collected 0.5 cm from the injury site and immediately cut into 1 × 1 × 1 mm^3^ pieces. These samples were fixed in 2.5% glutaraldehyde at 4 °C for 24 h. After washing with PBS, the samples were postfixed in 1% osmium tetroxide at 4 °C for 2 h and then dehydrated through a graded acetone series. The samples were then embedded in epoxy resin at 37 °C for 12 h and placed in a 65 °C oven for an additional 48 h.

### Bulk RNA sequencing

Neonatal and adult mice underwent T9–T10 spinal cord crush injuries, with tissues extracted from a 1-mm segment surrounding the uninjured spinal cord (sham group) and the injury site at 1, 3, and 7 dpi. For neonatal mice, spinal cord samples from each cage were pooled into one sample, while for adult mice, spinal cords from 3 to 4 individual mice were pooled into a single sample. Total RNA was isolated according to the manufacturers’ protocols. Samples were clustered using the cBot Cluster Generation System, and sequencing was performed on an Illumina NovaSeq 6000 platform using the PE150 mode. Quality control was performed to eliminate adapters, low-quality reads, and duplicates, resulting in clean reads mapped to the mouse reference genome (mm10) using Hisat2. Gene expression levels were quantified using fragments per kilobase of transcript per million mapped reads (FPKM). GSEA was performed using the clusterProfiler v4.9.3, with results visualized through GseaVis v0.0.9. GO analysis was performed using the clusterProfiler v4.9.3, with result visualization done via ggplot2 v3.3.6.

### GEO datasets of microarray and bulk RNA sequencing

We obtained annotated and pre-processed microarray data from the Gene Expression Omnibus (GEO) database. The detailed information for each GEO dataset is provided in Table S4.

### Differential gene analysis and heatmap generation

DEGs were detected and quantified using Limma v3.52.1. The significance threshold for differential expression at both tissue and cellular levels was set at a *P* value of <0.05, with an absolute average log_2_ fold change greater than 1.50. Heatmap and clustering analyses were performed to visualize and interpret gene expression patterns across samples. Clustering was conducted using the mfuzz algorithm implemented in ClusterGVis v0.0.9, which identified distinct clusters based on expression similarities. Sample annotations and heatmap generation were incorporated using the HeatmapAnnotation function from ComplexHeatmap v2.16.0.

### Thanatoset gene panel identification

A comprehensive list of pan-PCD genes was compiled by integrating data from GSEA, WIKI, Reactome, and Ferrdb databases and extensive literature review, resulting in 963 genes associated with 13 PCD types. DEGs from early injury phases (1, 3, and 7 dpi) in crush SCI were identified, and overlapping genes across contusion and hemisection models were determined using rank-sum tests. Protein–protein interactions (PPIs) of the Thanatoset were screened using the STRING database. Venn diagrams were generated using the EVenn online tool.

### Single-cell/nucleus suspension preparation and library construction

Spinal cord tissue was collected at 3 dpi from each group (sham, SCI, and SCI + WFA). For each group, spinal cord segments approximately 1 cm in length from the injury area were isolated from 3 to 4 mice for subsequent analysis. Single-cell suspensions (2 × 10^5^ cells/ml) were prepared in PBS and loaded onto a microwell chip using the Singleron Matrix Single Cell Processing System. scRNA-seq libraries were constructed according to the protocol of the GEXSCOPE Single Cell RNA Library Kits (Singleron). The libraries were diluted to 4 nM, pooled, and sequenced on the Illumina NovaSeq 6000 platform with 150-base pair paired-end reads.

### Single-nucleus suspension preparation and library construction

Spinal cord tissue was collected at 3 dpi from each group (sham, 3 dpi, and 7 dpi) in both adult and neonatal mice. For adult mice, the sampling method was identical to that described previously. For neonatal mice, spinal cord segments approximately 0.5 cm in length from the injury area were collected from a litter (minimum of 6 pups) for subsequent analysis. First, tissue samples were dissociated using the MobiSoultion Tissue Dissociation Kit to prepare single-cell suspensions. Then, the MobiCube Single Cell 3′ Reagent Kits User Guide (PN–S050400301) was used to construct the cDNA library, following protocols for cell counting, quality control, gel bead-in-emulsion generation, barcoding, cDNA amplification, and gene expression library construction [75].

Cell barcodes and unique molecular identifiers (UMIs) were extracted, and reads were mapped to the GRCm38 reference genome using STAR v2.6.1a. UMI and gene counts for each cell were obtained using featureCounts v2.0.1 and used to generate gene expression matrices for subsequent analysis. To remove doublets, the Find_doublet function was applied. This function uses the DoubletFinder v2.0.3 to perform doublet identification based on principal components analysis (PCA) with a specified number of dimensions (dim.usage = 25). After doublet removal, the datasets were merged into a single Seurat object using merge. Additional quality control steps were performed by calculating the percentage of mitochondrial genes (percent.mt) and hemoglobin genes (HB_percent). The final dataset was subsetted to retain cells with 300 to 10,000 features, mitochondrial content below 20%, and hemoglobin content below 0.1%. The processed data were saved for further analysis. For cell-type annotation, we combined the SingleR package with manual annotation based on prior literature to assign cell types.

### Gene set scoring and visualization for single-cell/nucleus datasets

We performed gene set scoring and visualization on scRNA-seq data using several R packages, including decoupleR v2.5.2 and irGSEA v2.1.5. First, the Thanatoset gene set was filtered to match the genes present in the Seurat object. The gene set was then used to perform gene set scoring with the irGSEA.score function, applying multiple methods, including AUCell v1.18.1, UCell v1.31, singscore v1.20.0, AddModuleScore function, and ssGSEA function. To determine the optimal threshold for distinguishing high and low Thanatoset scores, we used the AUCell_exploreThresholds function, which provides a histogram and automatically assigns cells based on the calculated threshold.

We calculated the slopes and confidence intervals for the AUC values across different experimental groups (sham, SCI). For each cell type, a linear regression model [*lm*(*AUC ~ Group*)] was fitted with AUC values as the dependent variable and the experimental groups as the independent variable. The coefficient for the group variable was extracted as the slope, and the confidence intervals for these slopes were calculated using the confint() function.

### Cell–cell communication analysis

Using CellChat v1.5.0, cell–cell communication was inferred with the single-cell transcriptomic dataset. Briefly, according to the expression of a ligand by one lineage and a receptor by another, potential ligand–receptor interactions were predicted. For each ligand–receptor pair, we obtained a *P* value and connection possibility for the likelihood of lineage specificity.

### WGCNA

Network analysis was conducted using WGCNA v1.72-1 to identify key gene modules associated with the adult and neonatal groups. WGCNA begins by selecting highly variable genes, filtering those with variance above the 25th percentile. A soft threshold power is then determined through scale-free topology analysis, targeting an *R*^2^ value greater than 0.85. Gene Significance and Module Membership metrics are calculated to assess the connection between individual genes, traits, and modules.

### Spatial transcriptomics

Raw sequencing data from the T9–T10 spinal cord transection of 8- to 9-week-old adult female mice were obtained from GSE256397. The matrices, along with corresponding tissue images, were imported into Seurat v4.3.0 for downstream analysis. Data normalization and scaling were performed using SCTransform to account for technical variability. For datasets involving multiple samples, integration was achieved using the FindIntegrationAnchors and IntegrateData functions to correct batch effects and enable comparative analyses. Spatial visualization was conducted using the SpatialFeaturePlot function to overlay gene expression data onto tissue images, highlighting specific genes, pathways, or clusters. Additionally, the AddModuleScore function was used to assess the spatial localization of the Thanatoset pathway.

### CMap analysis

To reprogram adult SCI microglial pan-PCD toward a neonatal-like state, we first downloaded the dataset of drug-responsive gene expression profiles from CMap (www.broad.mit.edu/cmap). First, we performed differential expression analysis using the FindAllMarkers function on 3 groups of microglia from adult mice post-SCI compared to one group of microglia from neonatal mice post-SCI. LogFC values for the Thanatoset gene set were extracted for further analysis. Second, CMap generated enrichment scores ranging from +100 to −100 for these perturbagens, ranked based on their similarities or dissimilarities to the input Thanatoset gene signatures. Based on the training set, the top 20 compounds with marked negative correlations were identified, and their correlation coefficients and *P* values were extracted from GSE182803 and GSE205037. Third, compounds that were markedly negatively regulated across all 3 comparisons (*P* < 0.05) were selected, and the top 5 most promising compounds were further filtered based on their biological safety profiles as reported in the literature. The framework for the identification of candidate drugs based on this drug-repositioning strategy is illustrated in Fig. 4A.

### Molecular docking simulations

To assess the interactions of 5 candidate compounds with 49 fully resolved mouse proteins from the Thanatoset, we performed molecular docking simulations using established computational methods [76]. The 3-dimensional (3D) structures of the 49 proteins were either retrieved from the Protein Data Bank for those lacking available structures. The candidate compounds were obtained from chemical databases (PubChem) and converted from 2D to 3D structures using Open Babel v3.1.1 and Chem3D v20.1. Ligand geometry was optimized, and partial atomic charges were assigned using appropriate force fields. Docking simulations were conducted using AutoDock Vina v1.1.2 and Glide v6.9, where the binding sites were either defined based on known active sites or determined via blind docking. The docking parameters, including grid box size and exhaustiveness, were set to ensure optimal docking results, and multiple poses were generated for each ligand–protein pair.

### In vitro microglial cell culture

BV2 microglial cells (SUNNCELL, Wuhan, China) were maintained in BV2-specific medium at 37 °C under 5% CO_2_. To induce pan-PCD, cells in the LPS + adenosine group were treated with 1 μg/ml LPS (#L6143, Sigma-Aldrich) for 24 h, followed by 500 μM adenosine (#A4036, Sigma-Aldrich) for 2 h. Cells in the WFA group were treated with WFA (0 to 500 nM) for 24 h.

### Cell viability assay

Cell viability was assessed using the Cell Counting Kit-8 (CCK-8) assay (Servicebio) following the manufacturer’s protocol. BV2 cells were seeded in 96-well plates overnight and treated with WFA (0 to 500 nM) for 24 h. Absorbance at 450 nm was measured using a microplate reader (BioTek, USA), and cell viability was calculated for each WFA treatment group.

### Flow cytometry

Microglia cells were isolated and filtered through a 70-μm cell strainer to obtain a single-cell suspension. Apoptosis was assessed using the Annexin V-FITC/PI Apoptosis Detection Kit (MCE) according to the manufacturer’s instructions. Cells were incubated with Annexin V-FITC (fluorescein isothiocyanate) and PI (propidium iodide), and samples were acquired using a BD LSRFortessa flow cytometer (BD Biosciences, USA). Data were analyzed using FlowJo software (v10.8.1) to quantify apoptotic cell populations.

### ELISA

Cytokine levels in cell culture supernatants and tissue lysates were measured using ELISA. Samples were centrifuged to remove debris, and supernatants were stored at −80 °C until analysis. Specific ELISA kits (TNF-α, IL-1β, IL-6, nerve growth factor, brain-derived neurotrophic factor; Invitrogen) were used following manufacturer protocols.

### Primary extraction and culture of DRG neurons and explants

DRG were isolated from P1 to P3 Sprague–Dawley rats. The heads of the pups were removed, and the skin and bones along the spine and thoracic/cervical vertebrae were incised using micro-scissors to expose the DRG bilaterally. For DRG neuron isolation, the tissue was digested with collagenase type IV and 0.25% trypsin for 1 h, and the reaction was stopped by adding DF-12 medium supplemented with fetal bovine serum (FBS). The mixture was centrifuged at 1,000 rpm for 5 min, and the pellet was resuspended in Neurobasal complete medium. The suspension was filtered through a 100-μm cell strainer and plated on matrix gel-coated culture plates. For DRG explants, the same procedure was followed, but the ganglia were not cut into pieces, and the digestion time was reduced to 5 min.

After treatment with vehicle, LPS + adenosine, or WFA, BV2 microglia were seeded into the upper chambers of a 24-well Transwell plate (Corning). The neurons or explants were plated into the lower chambers of the Transwell system, ensuring physical separation between the cells in the upper and lower chambers. The coculture system was maintained for 48 h at 37 °C under 5% CO_2_. The effects of these treatments on DRG neuron and explant health and growth were assessed. Neurite outgrowth was analyzed using Sholl analysis with Fiji software (v2.1.0) and the Sholl Analysis plugin.

### Culture of VSC4.1 motoneurons

VSC4.1 neurons (#EDWT0066, EditGene) were cultured in Dulbecco’s modified Eagle’s medium (DMEM) supplemented with 10% FBS and 1% penicillin/streptomycin at 37 °C under 5% CO_2_. BV2 microglial cells were seeded in the upper chambers of a 24-well Transwell plate (Corning), while VSC4.1 neurons were plated in the lower chambers. This setup allowed for the diffusion of soluble factors between microglia and neurons while preventing direct cell–cell contact. After 48 h of coculture under standard conditions, the effects of the treatments on VSC4.1 neurons were assessed.

### Western blotting

Proteins were extracted from microglia using radioimmunoprecipitation assay (RIPA) buffer supplemented with protease and phosphatase inhibitors. Protein concentrations were determined via the bicinchoninic acid (BCA) assay. Equal amounts of protein (20 to 30 μg) were separated by sodium dodecyl sulfate–polyacrylamide gel electrophoresis (SDS-PAGE) and transferred onto polyvinylidene fluoride membranes. Membranes were blocked with 5% nonfat milk in TBST (tris-buffered saline with 0.1% Tween 20) for 1 h at room temperature, followed by incubation overnight at 4 °C with primary antibodies: anti-GSDMD (#20770-1-AP, Proteintech), anti-GSDMD-N (#DF13758, Affinity), anti-Caspase-8 (#4790, Cell Signaling Technology), anti-Cleaved-CASP8 (#8592, Cell Signaling Technology), anti-CD68 (ab283654, Abcam), anti-TLR2 (ab209216, Abcam), rabbit anti-NF-κB p65 (Abcam, ab32536), rabbit anti-NF-κB p65 (phospho-S536, Abcam, ab76302), rabbit anti-IκBα (Abcam, ab325186), and rabbit anti-IκBα (phospho-S32, Abcam, ab92700). After washing with TBST, membranes were incubated with horseradish peroxidase-conjugated secondary antibodies for 1 h at room temperature. Protein bands were visualized using an enhanced chemiluminescence solution (Millipore) and imaged with an Amersham Imager 600 Station. Protein expression levels were quantified by densitometric analysis of immunoblot bands using ImageJ software, with target protein signals normalized to the corresponding β-actin signals prior to statistical analysis.

### Statistical analysis

Statistical analyses were conducted using R (v4.2.0) and GraphPad Prism (v8.0.2). Data are presented as mean ± standard error of the mean (SEM). For comparisons between 2 groups, a 2-tailed *t* test was used for normally distributed data, and the Wilcoxon rank-sum test was applied for non-normally distributed data. For multiple group comparisons, 1-way or 2-way analysis of variance (ANOVA) followed by Tukey’s post hoc test was performed. Statistical significance was set at *P* < 0.05. All experiments were performed with at least 3 biological replicates unless otherwise specified.

## Data Availability

The transcriptome data generated in this study have been deposited in the GEO database under the accession number GSE285895. Additional materials or datasets can be obtained from the corresponding author upon reasonable request.
