# Supplementary material for: Neonatal-Inspired Reprogramming of Microglial Pan-Programmed Cell Death Enhances Regeneration in Adult Spinal Cord Injury
Source: Research (Wash D C). 2025 Jul 2;8:0759. doi: 10.34133/research.0759 (PMC12220820; doi:10.34133/research.0759)
Supplement: Supplementary 1 — Figs. S1 to S10 Tables S1 to S4 [file research.0759.f1.zip › supplymentary.docx]

**Supplementary Materials**


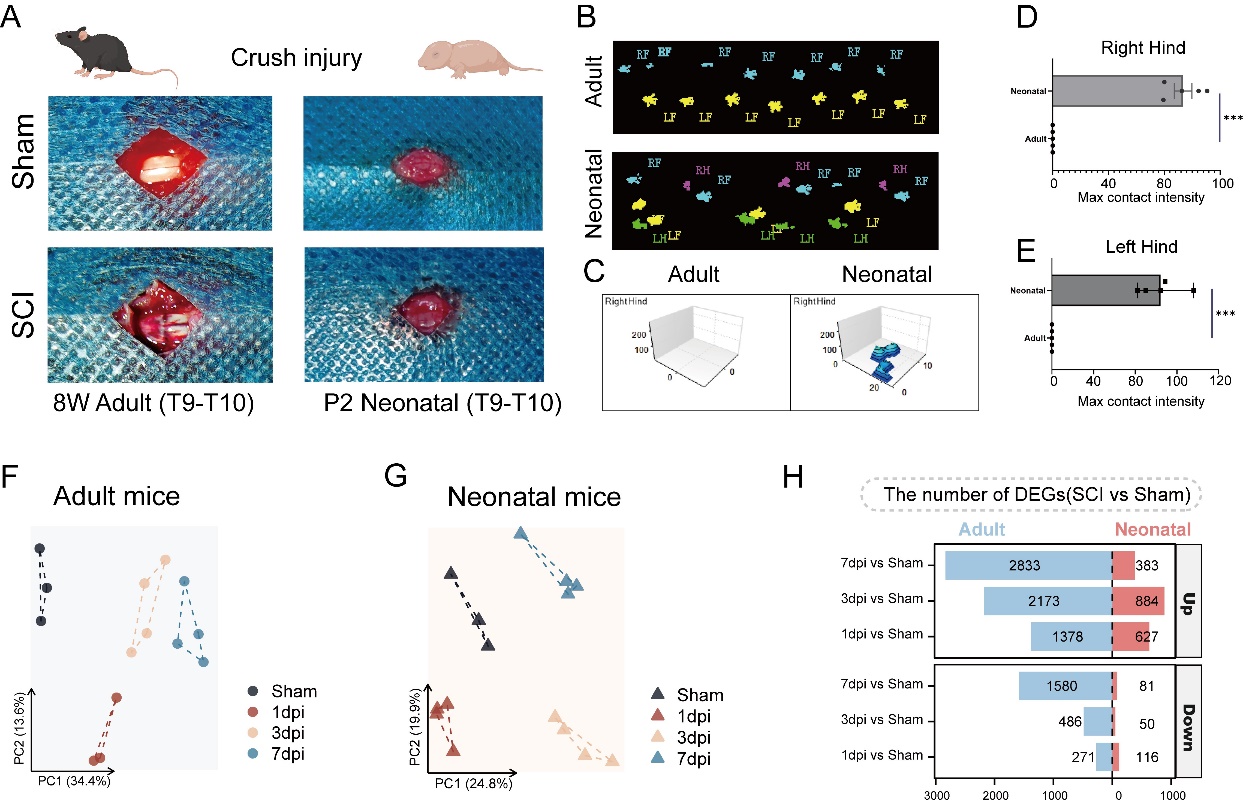


**Fig. S1.** Establishment and Characterization of the T9-T10 SCI Crush Model in Neonatal and Adult Mice. (A) Images showing the T9-T10 crush SCI model in 8-week-old adult (left) and postnatal day 2 (P2) neonatal mice (right) in both Sham and SCI conditions. (B) Representative CatWalk gait trajectories of mice in each group at 42 dpi. (C) Representative 3D stress distribution diagrams at 42 dpi. (D-E) Quantitative analysis of maximum contact intensity for the right (D) and left (E) hindlimbs in neonatal and adult mice (n = 5). (F-G) PCA plots of bulk RNA-sequencing data for adult (F) and neonatal (G) mice across sham, acute (1 dpi), intermediate (3 dpi), and subacute (7 dpi) phases. (h) Bar graphs showing the number of DEGs in SCI vs. Sham groups at 1, 3, and 7 dpi for adult (blue) and neonatal (red) mice. Statistical analysis was conducted using a two-tailed t-test [(D) and (E)]. ns (not significant), ****P* < 0.001.


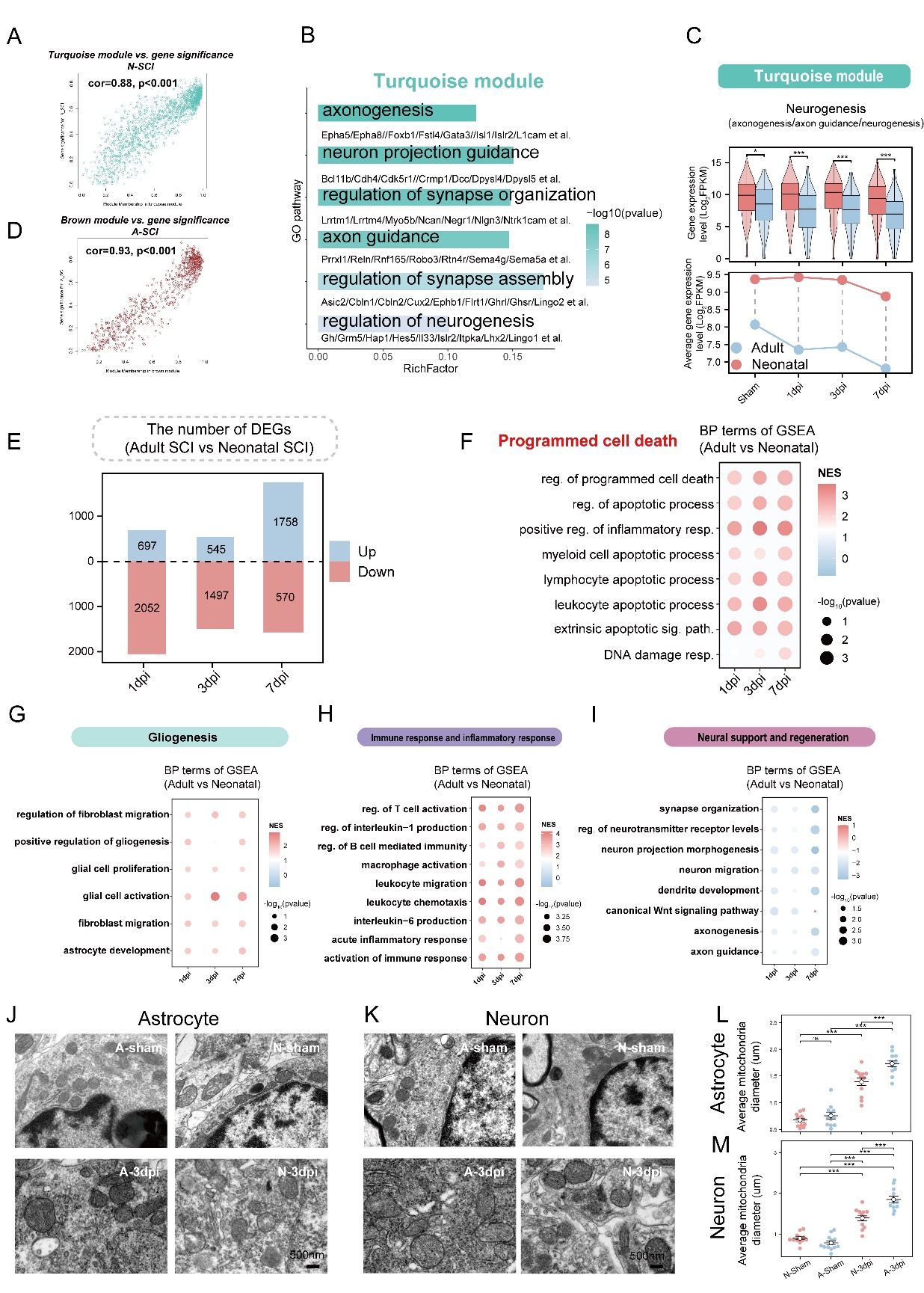


**Fig. S2.** Functional and Morphology Differences Between Adult and Neonatal Mice Post-SCI. (A) Scatter plot showing gene significance in neonatal SCI correlations with turquoise module (*cor* = 0.88, *P* < 0.001) from WGCNA. (B) GO enrichment analysis of the turquoise module (*P* < 0.01). (C) Scatter plot showing gene significance in adult SCI correlations with brown module (*cor* = 0.93, *P* < 0.001). (D) Violin plots showing the average expression (Log_2_FPKM) of neurogenesis-related genes in the turquoise module across time points (upper panel) and their comparison between neonatal and adult SCI groups (lower panel). (E) Bar graph displaying the number of DEGs (upregulated and downregulated) in adult SCI compared to neonatal SCI at 1, 3, and 7 dpi. (F-I) GO terms of GSEA for: F PCD-related pathways, G gliogenesis, H immune and inflammatory responses, and I neural support and regeneration, comparing adult and neonatal SCI. (J-K) Representative TEM images of astrocytes (J) and neurons (K) from lesion rims of adult and neonatal mice, showing differences in mitochondrial morphology at 3dpi. (L-M) Quantification of mitochondrial diameter in astrocytes (L) and neurons (M), highlighting better mitochondrial preservation in neonatal mice (n = 12). Statistical analysis was conducted using a Wilcoxon rank-sum test (C) or one-way ANOVA with Tukey (L and M). ns (not significant), **P* < 0.05, ****P* < 0.001.


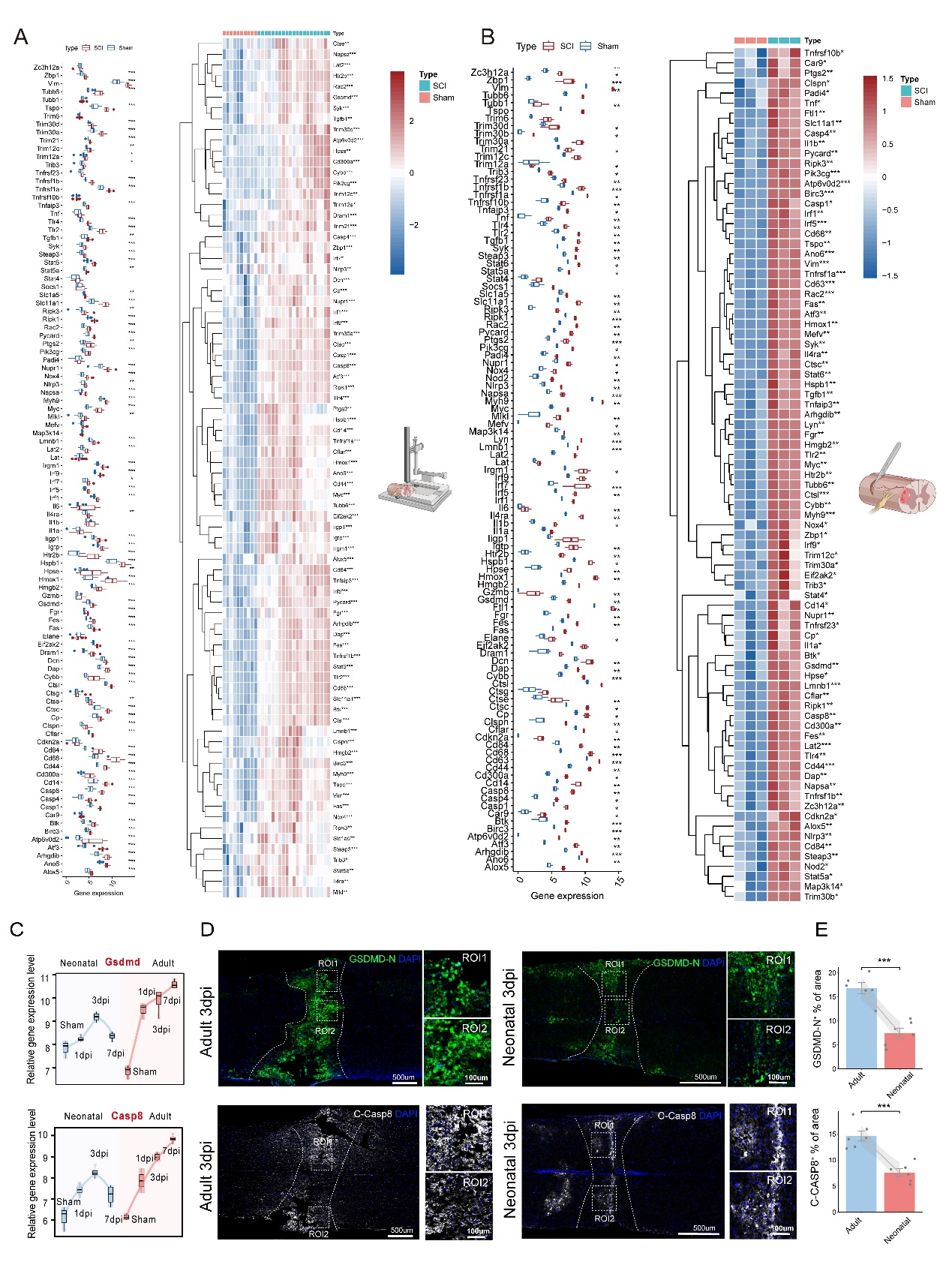


**Fig. S3.** Construction and Validation of the Thanatoset Gene Panel. (A) Identification of pan-PCD genes in adult mouse contusion models using rank-sum tests based on GSE5296 and GSE47681 datasets. (B) Identification of pan-PCD genes in adult mouse hemisection models using rank-sum tests based on the GSE171441 dataset. (C) Line graph showing the average mRNA expression levels of *Gsdmd* and *Casp8* in different groups of neonatal and adult mice. (D) Representative images of GSDMD-N and Cleaved-CASP8 (C-Casp8) staining in the lesion core of neonatal and adult mice at 3 dpi. (E) Quantification of GSDMD-N and C-Casp8-positive area per section, as shown in (D), across groups (n = 6). Statistical analysis was conducted using Wilcoxon rank-sum test (A and B) or a two-tailed *t*-test (E). ns (not significant), **P* < 0.05, ***P* < 0.01, ****P* < 0.001.

**
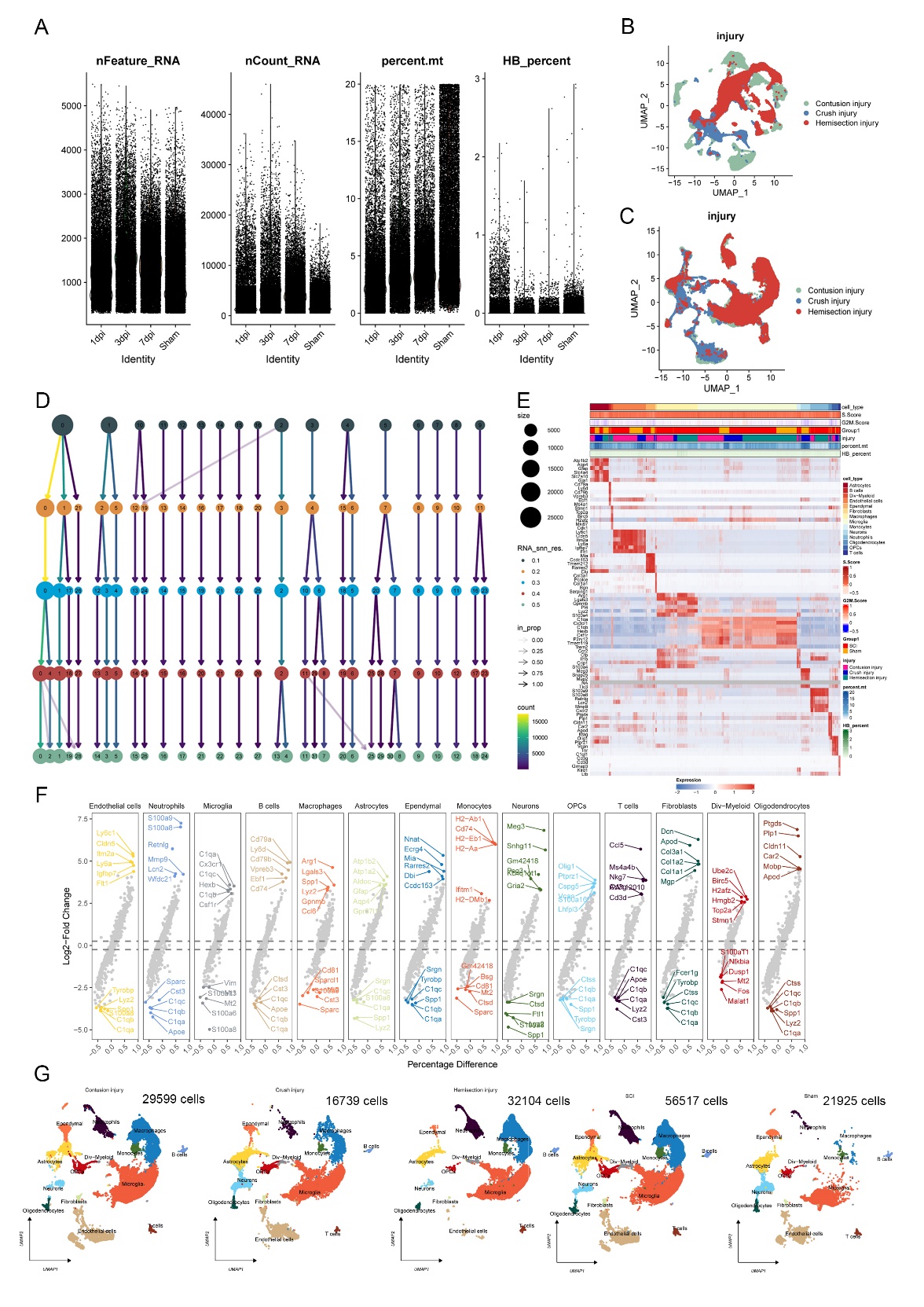
**

**Fig. S4.** Workflow for scRNA-seq Analysis in the Injured Adult Mouse Spinal Cord. (A) Quality control metrics for single-cell libraries from injured and uninjured mouse spinal cords, including *nFeature*, *nCount*, mitochondrial percentage (*mt_percent*), and hemoglobin percentage (*HB_percent*) per cell. (B-C) UMAP visualization of adult mouse contusion, crush, and hemisection SCI models before (B) and after (C) batch effect correction. (D) Number of identified cell subpopulations across resolutions ranging from 0.1 to 0.5. (E) Heatmap of classical marker genes for the 14 identified cell types. (F) Volcano plots showing the top six upregulated and downregulated genes for each of the 14 cell types. G UMAP visualization illustrating the distribution of cells across different SCI models (contusion, crush, and hemisection) and groups (SCI and Sham).

**
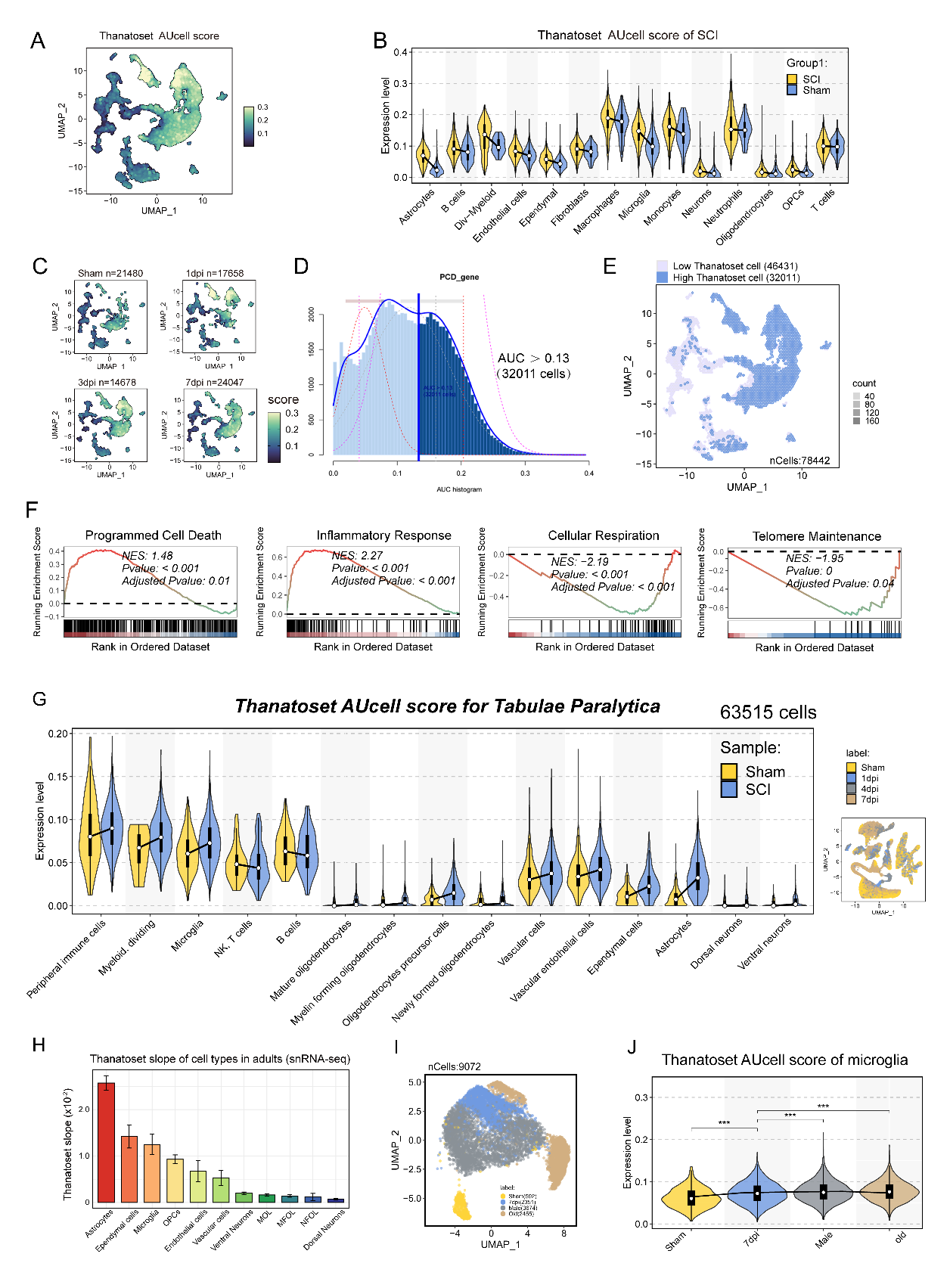
**

**Fig. S5.** Evaluation of Pan-PCD Levels in Single Cells by scRNA-seq and snRNA-seq Using the Thanatoset Gene Panel. (A) UMAP visualizations showing Thanatoset scores in 78,442 cells in scRNA-seq. (B) Comparison of Thanatoset scores across different experimental groups (Sham, SCI) for various cell types in scRNA-seq. (C) UMAP visualizations showing Thanatoset scores across different time points (Sham, 1 dpi, 3 dpi, and 7 dpi). (D) Threshold determination for high and low Thanatoset score cells using the AUCell scoring tool (cut-off: 0.13). (E) UMAP visualization differentiating cells with high versus low Thanatoset scores. (F) GSEA comparing high versus low Thanatoset score cells to identify key GO pathway enrichments. (G) Violin plot illustrating the increase in Thanatoset scores across all cell types post-injury in snRNA-seq, based on the Tabula Paralytica dataset. The left panel shows UMAP visualizations of 63,515 cells from Sham, 1 dpi, 4 dpi, and 7 dpi snRNA-seq data. (H) Slope of Thanatoset scores for different cell types post-SCI in adult mice relative to the Sham group in snRNA-seq, modeled using a linear model (*model <- lm(AUC ~ Group)*). (I) UMAP visualization highlighting microglial cells within the 9,072-cell dataset across Sham and 7 dpi groups under different conditions (vehicle, male, old). (J) Violin plot illustrating differences in Thanatoset scores across experimental-microglia groups. Statistical analysis was conducted using one-way ANOVA with Tukey (J). ****P* < 0.001.

**
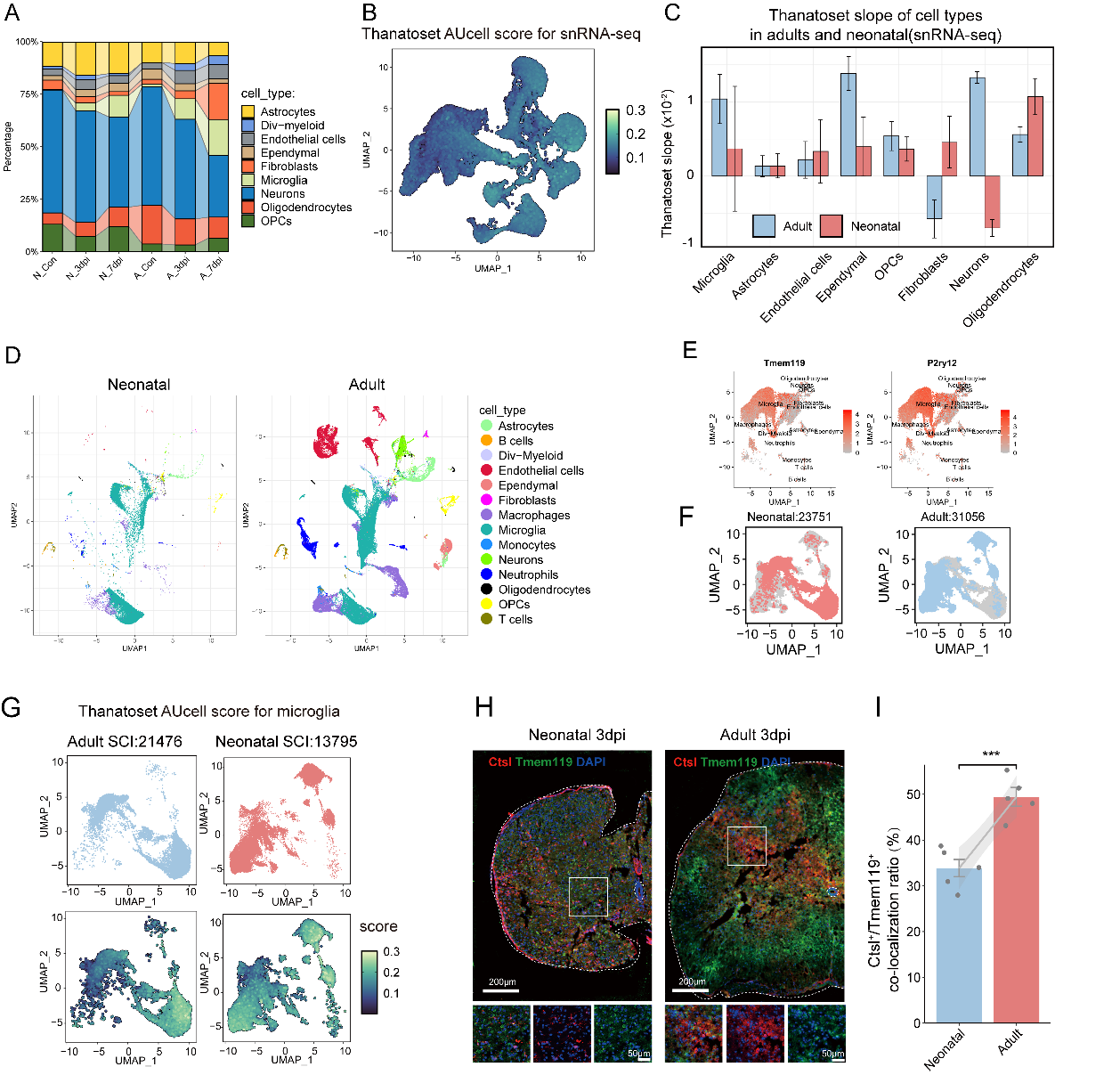
**

**Fig. S6.** Evaluation of Pan-PCD Levels in Microglia After SCI in Adult and Neonatal Mice. (A) Sankey diagram illustrating the flow and distribution of each cell type across the six experimental groups in snRNA-seq of adult and neonatal mice, encompassing 52,021 nuclei. (B) UMAP visualizations showing Thanatoset scores in 52,021 nuclei in scRNA-seq. (C) Bar plot comparing the slope of Thanatoset scores for different cell types between neonatal and adult mice post-SCI relative to Sham groups. (D) Annotation of neonatal spinal cord scRNA-seq data using Symphony software based on adult spinal cord scRNA-seq data (Fig.3a). (E) UMAP visualization showing the expression of classic microglial marker genes *Tmem119* and *P2ry12* in neonatal scRNA-seq data. (F)UMAP visualization of all microglia from 23,751 neonatal cells (Sham, 2 dpi, and 5 dpi) and 31,056 adult cells (Sham, 1 dpi, 3 dpi, and 7 dpi). (G) UMAP visualizations showing Thanatoset scores in adult microglia after SCI (21,476 cells) and neonatal microglia after SCI (13,795 cells). (H) Representative images of CTSL and TMEM119 staining in the lesion rims (0.5 mm from the injury area) of neonatal and adult mice at 3 dpi. I Quantification of CTSL^+^ and TMEM119^+^ cells co-localization ratios, as shown in H, across groups (*n* = 5). Statistical analysis was conducted using a two-tailed *t*-test (I). *** *P* < 0.001.


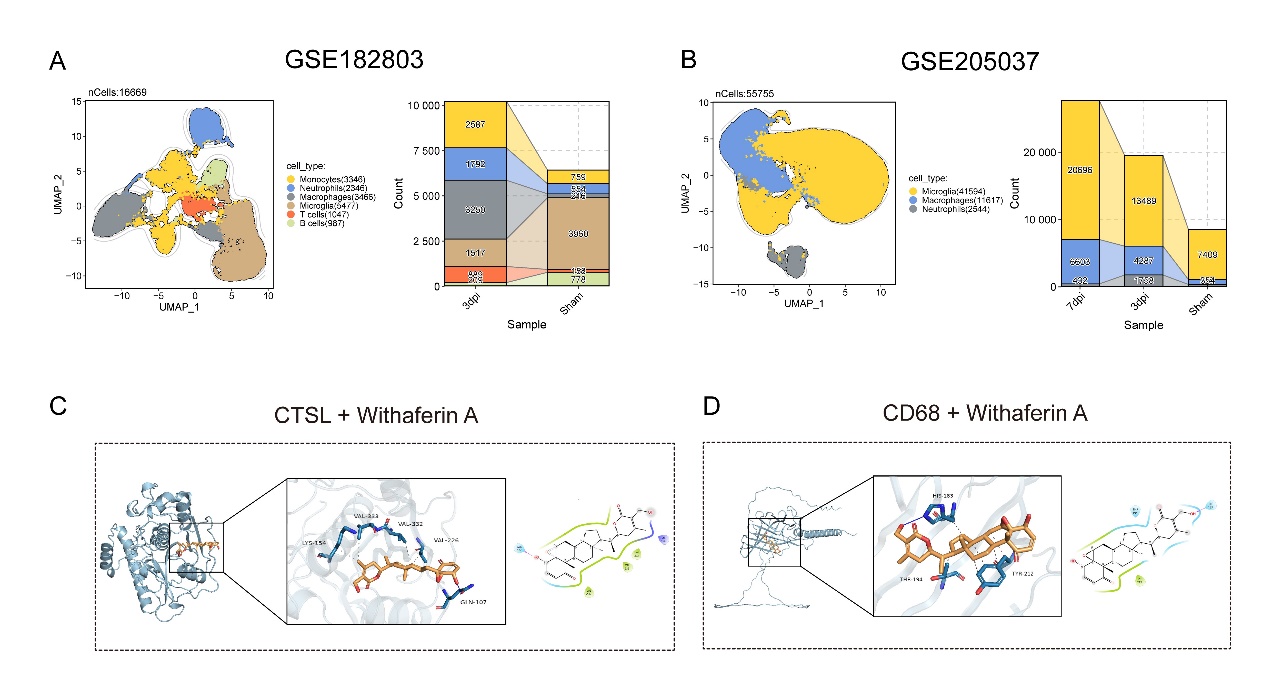


**Fig. S7.** Validation of Thanatoset-Based Pan-PCD Inhibitor Screening in Microglia Post-SCI. (A) UMAP visualization of cells from the GSE182803 dataset (1,517 Microglia) showing cell type distribution across injury phases (Sham, 1 dpi, 3 dpi, and 7 dpi). The accompanying bar chart displays sample counts per condition. (B) UMAP visualization of cells from the GSE205037 dataset (34,185 cells), highlighting cell types (monocytes, microglia, neutrophils) and their proportions across injury phases. (C-D) Molecular docking visualization of Withaferin A binding to key Thanatoset proteins: CTSL (C) and CD68 (D). (C) Withaferin A forms hydrophobic interactions with VAL333, LYS154, VAL332, and VAL226 of the CTSL protein, and its OH group forms a hydrogen bond with GLN107. (D) Withaferin A forms hydrophobic interactions with TYR212 and THR194 of the CD68 protein, and its OH group forms a hydrogen bond with HIS183. Proteins are displayed as cartoons, with blue stick models representing amino acid residues, yellow representing small molecules, solid blue lines for hydrogen bonds, gray dashed lines for hydrophobic interactions, and green dashed lines for π-π stacking interactions.


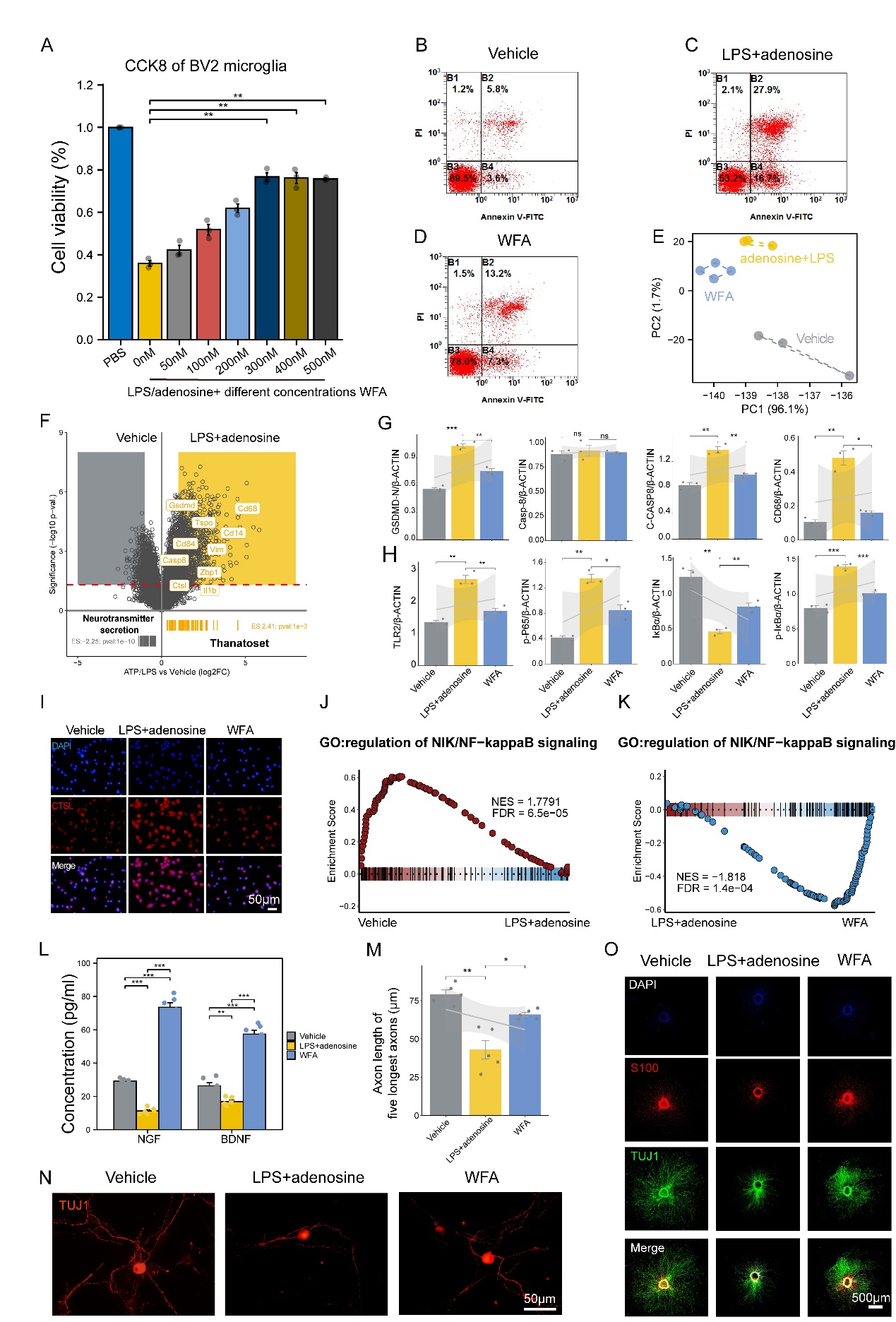


**Fig. S8.** WFA Restores Cell Viability and Reduces Pan-PCD in LPS+adenosine-Treated Microglia. (A) CCK8 assay measuring cell viability of LPS+adenosine-treated BV2 microglia exposed to varying WFA concentrations (0–500 nM). (B-D) Representative flow cytometry images of microglial apoptosis: B Vehicle group. C LPS+adenosine group, showing increased apoptosis. D WFA group, demonstrating reduced apoptosis following WFA treatment. (E) PCA plot comparing transcriptional profiles of Vehicle, LPS+adenosine, and WFA groups. (F) Volcano plot comparing gene expression between LPS+adenosine-treated microglia and Vehicle groups. Specific GSEA pathways enriched in each group (Neurotransmitter secretion and Thanatoset) are displayed below. (G) Quantification of protein expression level of NF-κB pathway components (GSDMD-N, CASP8, Cleaved-CASP8, and CD68) across groups (n = 3) (H) Quantification of protein expression level of NF-κB pathway components (TLR2, p-P65, IκBα, and p-IκBα) across groups (n = 3) (I) Representative immunofluorescence images showing CTSL expression (red) in microglia across Vehicle, LPS+adenosine, and WFA groups. (J-K) GSEA enrichment plot demonstrating significant upregulation of the "regulation of NIK/NF-κB signaling" pathway in LPS+adenosine-treated microglia compared to the Vehicle group (J) and significant downregulation of the "regulation of NIK/NF-κB signaling" pathway in WFA-treated microglia compared to the LPS+adenosine group (K). (L) ELISA analysis of NGF and BDNF levels in the supernatants of microglial cultures, showing increased secretion of neurotrophic factors in the WFA-treated group. (M) Quantification of axon lengths of the five longest axons (TUJ1-stained DRG neurons), indicating enhanced axonal growth in DRG neurons co-cultured with WFA-treated microglia. (N) Representative images of TUJ1-stained DRG neurons in co-culture with microglia from Vehicle, LPS+adenosine, and WFA groups. (O) Representative immunofluorescence images of DRG explants co-stained for DAPI (nuclei, blue), S100 (glia marker, red), and TUJ1 (axon marker, green). Statistical analysis was conducted using one-way ANOVA with Tukey (K, L). **P* < 0.05, ***P* < 0.01, ****P* < 0.001.


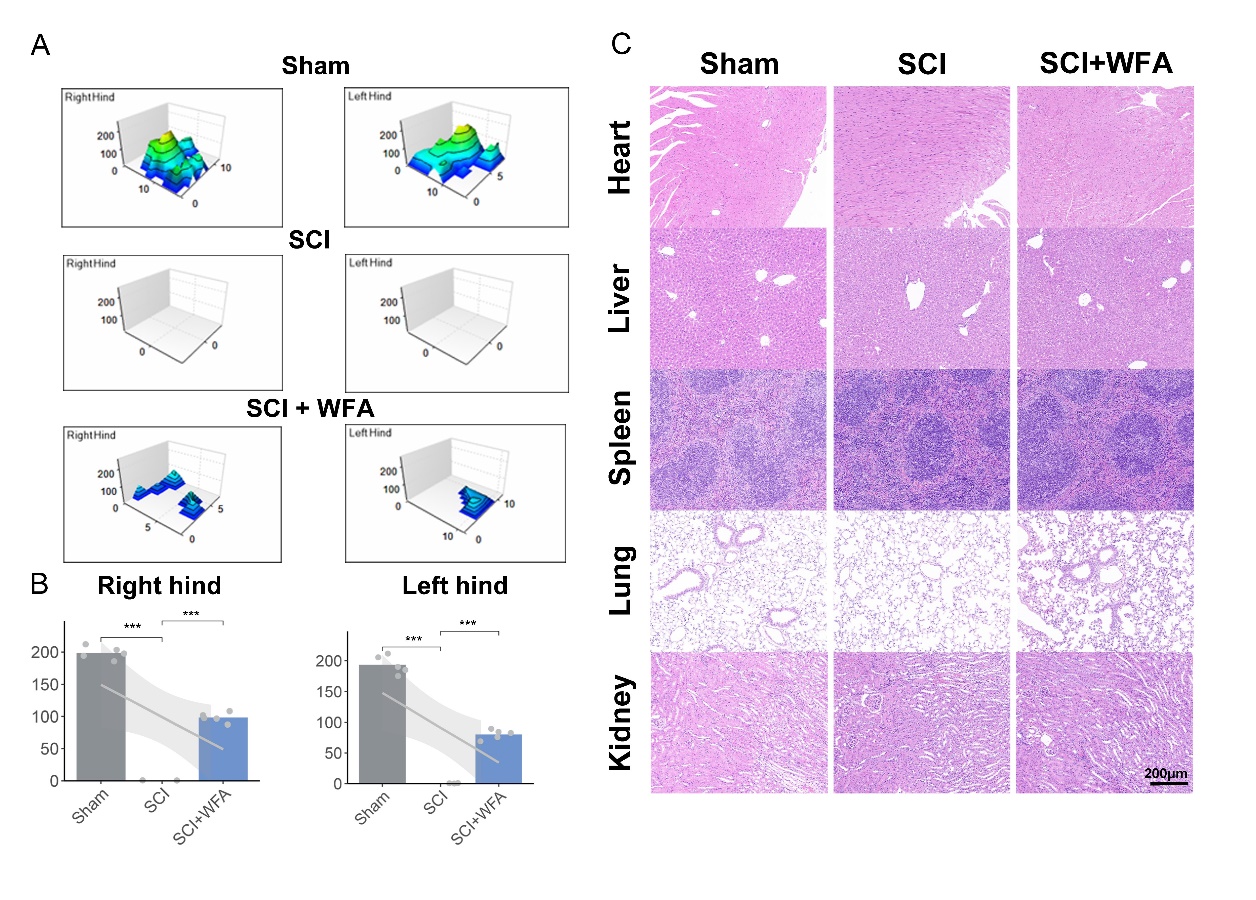


**Fig. S9.** Functional and Safety Evaluation of WFA Treatment Post-SCI. (A) Representative 3D plots from CatWalk analysis showing hind limb pressure distribution in Sham, SCI, and SCI+WFA groups. (B) Quantification of hind limb plantar pressure (right and left) from CatWalk analysis, demonstrating the effect of WFA on pressure recovery (n=5). (C) Representative H&E staining images of major organs (heart, liver, spleen, lung, and kidney) from Sham, SCI, and SCI+WFA groups, indicating no significant toxicity of WFA treatment. Statistical analysis was conducted using one-way ANOVA with Tukey (B). **P* < 0.001.


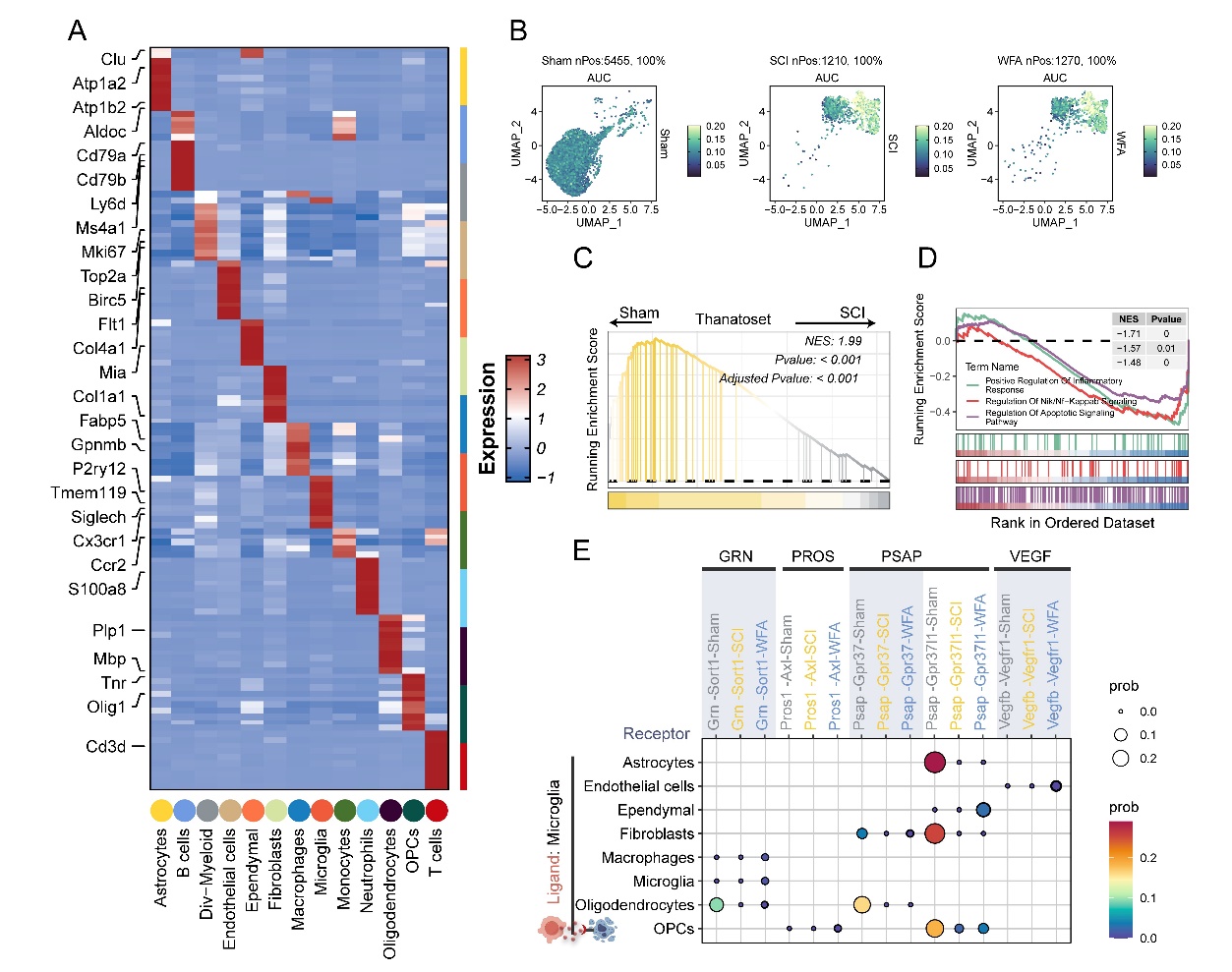


**Fig. S10.** Validation of WFA Effects on Microglial Pan-PCD and Intercellular Communication Post-SCI. (A) Heatmap showing the expression levels of marker gene across 13 cell types. (B) UMAP visualization of microglial Thanatoset scores for Sham, SCI, and SCI+WFA groups. (C) GSEA plot comparing the Thanatoset gene set between the SCI group and the Sham group, demonstrating significant upregulation of Thanatoset in microglia from the SCI group compared to the Sham group. (D) GSEA enrichment plots showing WFA-mediated suppression of NF-κB signaling, apoptosis regulation, and inflammatory response pathways in microglia post-SCI. (E) Dot plot illustrating ligand-receptor interactions mediated by microglia (as ligands) across groups (Sham, SCI, SCI+WFA) for neuroprotective signaling pathways GRN, PSAP, and VEGF. Bar color and dot size indicate cell interaction probability.

**Table S1.**

Gene Sets for 13 Types of Programmed Cell Death: Sources and Summary

**Table S2.**

Composition of the Thanatoset Gene Set: 68 Genes and Their Functions

**Table S3.**

Biochemical Properties of the Top 5 Pan-PCD Modulatory Compounds.

**Table S4.**

Detailed Information and Sources of Datasets Used in the Study.
